# The Essential Function of *B. subtilis* RNase III Is to Silence Foreign Toxin Genes

**DOI:** 10.1371/journal.pgen.1003181

**Published:** 2012-12-27

**Authors:** Sylvain Durand, Laetitia Gilet, Ciarán Condon

**Affiliations:** CNRS UPR 9073 (affiliated with Université Paris Diderot, Sorbonne Paris Cité), Institut de Biologie Physico-Chimique, Paris, France; University of Geneva Medical School, Switzerland

## Abstract

RNase III–related enzymes play key roles in cleaving double-stranded RNA in many biological systems. Among the best-known are RNase III itself, involved in ribosomal RNA maturation and mRNA turnover in bacteria, and Drosha and Dicer, which play critical roles in the production of micro (mi)–RNAs and small interfering (si)–RNAs in eukaryotes. Although RNase III has important cellular functions in bacteria, its gene is generally not essential, with the remarkable exception of that of *Bacillus subtilis*. Here we show that the essential role of RNase III in this organism is to protect it from the expression of toxin genes borne by two prophages, Skin and SPβ, through antisense RNA. Thus, while a growing number of organisms that use RNase III or its homologs as part of a viral defense mechanism, *B. subtilis* requires RNase III for viral accommodation to the point where the presence of the enzyme is essential for cell survival. We identify *txpA* and *yonT* as the two toxin-encoding mRNAs of Skin and SPβ that are sensitive to RNase III. We further explore the mechanism of RNase III–mediated decay of the *txpA* mRNA when paired to its antisense RNA RatA, both *in vivo* and *in vitro*.

## Author Summary

RNase III–related enzymes play key roles in cleaving double-stranded RNA throughout biology. Some of the best-known enzymes are RNase III itself, involved in ribosomal RNA maturation and mRNA turnover in bacteria, and Drosha and Dicer, which catalyze the maturation of micro (mi)–RNAs and small interfering (si)–RNAs in eukaryotes. Although RNase III has important cellular functions in bacteria, its gene is generally not essential, with the remarkable exception of that of *Bacillus subtilis*. In this paper, we show that the essential role of RNase III in this organism is to protect it from the expression of toxin genes borne by two prophages, through antisense RNA. *B. subtilis* is thus one of a growing number of organisms that uses RNase III or its homologs as part of viral defense or viral accommodation mechanisms, in this case to the point where the presence of the enzyme is essential for cell survival.

## Introduction

Ribonuclease III is a key enzyme for double-stranded (ds) RNA processing reactions in both bacterial and eukaryotic systems. In bacteria, it is best known for its role in ribosomal RNA maturation [1] and more recently has been shown to be involved in the regulation by small RNAs [2]–[4]. The enzyme was first identified and characterized for its roles in phage RNA (f2, T7 and lambda) processing in *E. coli* (for recent review, see [5]). Recently, RNase III has shown to be involved in bacterial gene silencing by processing CRISPR RNAs, generated as part of a host defense mechanism against phage DNA in many species [6]. In eukaryotes, enzymes with RNase III domains, such as Dicer and Drosha, play fundamental roles in the processes of RNA interference and in the generation of microRNAs [7], [8].

The recent influx of high resolution RNA transcriptome data has revealed a high level of antisense RNA transcription in many species and this has further stimulated interest in how cells deal with dsRNA on a larger scale. A recent study in *Staphylococcus aureus*, showed a relatively high level of pervasive transcription that is removed by RNase III [9]. Antisense RNAs to a large proportion of coding RNAs have also been revealed in *Helicobacter pylori*, *Synechocystis* and other organisms [10]–[12]. RNase III is the most likely candidate for controlling the level of dsRNA in these systems.

It has long been a mystery why RNase III, encoded by the *rnc* gene, is essential in *B. subtilis* [13]. It is possible to delete *rnc* in *E. coli* and other bacteria [14]–[16], although such mutations are often accompanied by a decrease in growth rate. Within the Firmicutes, it has been shown that the *rnc* gene is non-essential in *S. aureus* [2]. Furthermore, an RNase III-encoding gene is naturally lacking from *Deinococcus radiodurans* [17] and throughout the archaeal kingdom, although many archaea possess an enzyme of analogous function, called bulge-helix-bulge (BHB) or splicing endonuclease [18]. In eukaryotes, it has been shown that it is possible to delete the RNase III gene *rnt1* in yeast, albeit with severe growth defects [19]. The essential role of RNase III in *B. subtilis* is not related to its function in rRNA metabolism or in maturation of the scRNA, part of the essential 4.5S particle involved in co-translational insertion of proteins in the cellular membrane [13]. Indeed, only trace amounts of 30S rRNA precursor, much lower than in *E. coli*, are observed in the absence of RNase III in *B. subtilis*. This indicates that the enzymes that catalyze the final steps of rRNA maturation, RNase J1 [20], Mini-III [21] and RNase M5 [22], function efficiently without prior RNase III action.

We recently performed a tiling array analysis of *B. subtilis* strains depleted for RNase III and observed a surprisingly minor contribution of RNase III to the stability of many known antisense RNAs [23]. Furthermore, the effect of RNase III-depletion on many of the specific mRNAs tested was shown to be at the transcriptional level. The whole SigW regulon was up-regulated in this way under conditions of RNase III-deficiency, for example. One of the few RNAs tested that did show an effect on mRNA stability was the *txpA* mRNA. The *txpA* gene encodes a short (59 amino acids) hydrophobic peptide that causes cell lysis when overexpressed in *B. subtilis* [24]. It is part of a type I toxin/antitoxin (TA) system. TA systems were initially discovered as part of plasmid and transposon maintenance mechanisms and have more recently been found to be widespread on chromosomes, where they are thought to play important roles in adaptive responses to stress, including phenomena such as bacterial persistence and programmed cell death (for reviews, see [25], [26]). Typically, the toxin is a relatively stable molecule that targets some fundamental cellular function, such as membrane integrity, DNA replication or provokes RNA degradation, while the antitoxin is an unstable entity that needs to be constantly synthesized to counteract the toxin. In type II TA systems, both toxin and antitoxins are proteins, coded in an operon under control of a single promoter, whereas in type I TA systems, the antitoxin is an antisense (as) RNA, expressed from its own promoter on the opposite strand to the toxin gene (for recent review, see [27]). The antitoxin for the *txpA* mRNA is the RatA RNA and the 3′ ends of the two RNAs overlap [24]. Overexpression of RatA leads to degradation of the *txpA* toxin RNA, by an unknown mechanism. In this study, we show that the essential role of RNase III in *B. subtilis*, is the degradation of two type I toxin-encoding mRNAs, the *txpA* mRNA and the *yonT* mRNA from the Skin and SPβ prophages, respectively. In the absence of these two prophages or these two toxin-encoding mRNAs, deletion of the RNase III gene is possible and the growth rate of the resulting strains is hardly affected. We probe the role of RNase III in the mechanism of RatA mediated degradation of *txpA* mRNA both *in vivo* and *in vitro*.

## Results

### Some toxin-encoding mRNAs are stabilized in RNase III–depleted strains

**Figure 1:**
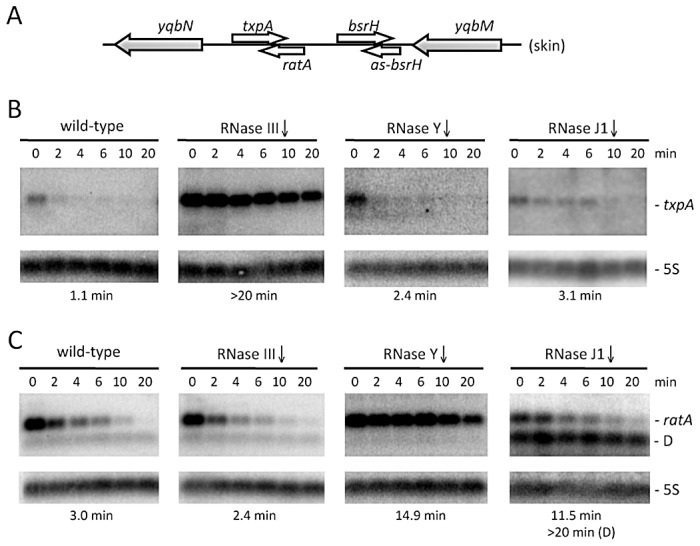
The *txpA* and RatA RNAs are stabilized in strains depleted for RNase III and RNase Y, respectively. (A) Chromosomal context of the *txpA*/RatA toxin/antitoxin cassette present in the Skin prophage. (B) and (C) Northern blots performed on RNAs isolated at times (min) after rifampicin addition (150 µg/ml) in strains depleted for RNase III (CCB288), RNase Y (CCB294) and RNase J1 (CCB034), probed for *txpA* and RatA, respectively. Northerns were re-probed for 5S rRNA (5S) for normalization. Half-lives are given below each panel. The band labeled D in panel C (RNase J1) is a degradation intermediate of RatA. Note that, in our hands, the *txpA* mRNA is about 45 nts longer than that proposed in [24] and consistent with the presence of a Rho-independent transcription terminator ∼270 nts from the mapped transcription start site. The overlap between RatA and *txpA* is predicted to be ∼120 nts.

In a recently performed analysis of *B. subtilis* strains depleted for the essential ribonucleases RNase Y, J1 or III, we noticed a significant increase in the steady-state levels of the type I toxin mRNA *txpA* in strains depleted for RNase III, while the RatA antitoxin RNA levels were elevated upon depletion of the single-strand specific RNase Y [23]. This was an intriguing result, because we had anticipated that the levels of both sense and antisense partners would be sensitive to the double-strand specific enzyme RNase III. We confirmed the results of the tiling array experiment by Northern blot analysis at times after the addition of rifampicin to inhibit new transcription initiation and showed that the increased expression in both cases was due to increased RNA stability (Figure 1). In an RNase J1 mutant, a degradation intermediate of the RatA asRNA accumulated. To determine whether this was a general phenomenon, we examined the expression patterns of two other suspected type I toxin/antitoxin cassettes, *bsrH/as-bsrH*, which lies adjacent to *txpA/RatA* on the chromosome, and *bsrG/as-bsrG(SR4)*, which has recently been studied by the Brantl group [28]. In both cases, the asRNA was stabilized in strains depleted for the single-strand specific enzyme RNase Y (Figures S1 and S2). However, while the *bsrG* toxin mRNA was significantly stabilized in the RNase III mutant, as seen by Jahn et al. [28], the *bsrH* toxin mRNA was not. Rather, it showed increased levels in the strain depleted for RNase J1. Thus, even among related type I TA systems, turnover mechanisms differ.

### The RNase III gene can be inactivated in strains lacking Skin and SPβ prophages

**Figure 2:**
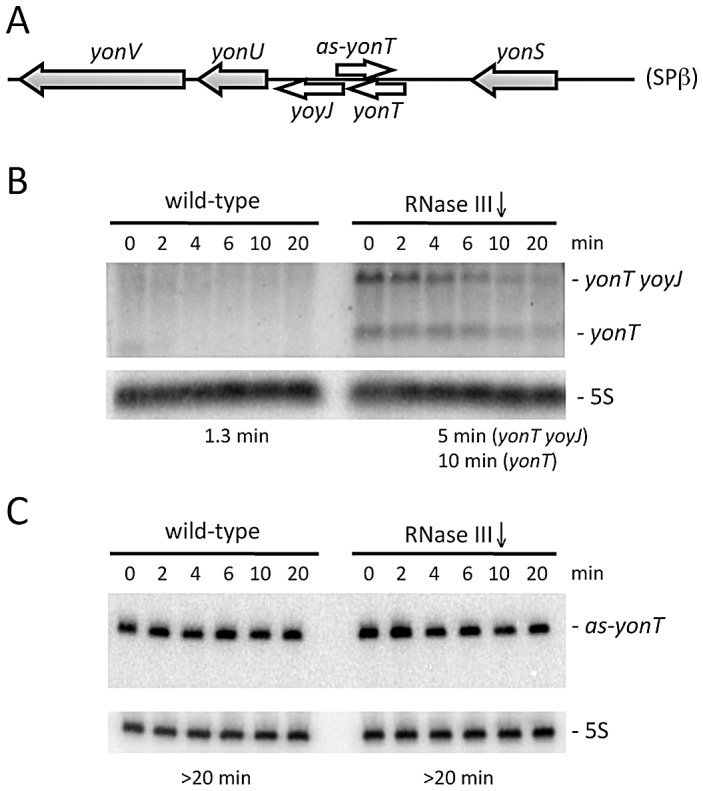
The *yonT* mRNA is stabilized in strains depleted for RNase III. (A) Chromosomal context of the *yonT/as-yonT* toxin/antitoxin cassette present in the SPβ prophage. (B) and (C) Northern blots performed on RNA isolated at times (min) after rifampicin addition in strains depleted for RNase III (CCB288), probed for *yonT* and as-yonT, respectively. The *yonT* probe was a uniformly ^32^P-labeled riboprobe, synthesized using a PCR fragment containing a T7 promoter as a template (oligos CC1101/1102; Table S2). The blots were re-probed for 5S rRNA (5S) for normalization. Half-lives are given below each panel.

The fact that RNase III is essential in *B. subtilis*, while the *rnc* gene can be deleted in other species, suggests that the *B. subtilis* enzyme has a specific function that explains why it cannot be removed. We wondered whether this essential function might be related to its role in toxin mRNA turnover. Both the *txpA/RatA* and *bsrH/as-bsrH* cassettes belong to the prophage known as Skin, while the *bsrG/as-bsrG(SR4)* pair is found on the SPβ prophage. SPβ also contains a second known type I TA system named *yonT/as-yonT* [27] and a TA pair that could be classified as type II, consisting of the precursor for the bacteriocidal sublancin peptide SunA and its immunity protein SunI [29]. Both of these toxin mRNAs are over-expressed in RNase III depleted strains, *yonT* through increased mRNA stability and *sunA* through higher transcriptional levels (Figure 2 and Figure S3). The as-yonT transcript is highly stable regardless of whether RNase III is present or not (Figure 2C).

**Figure 3:**
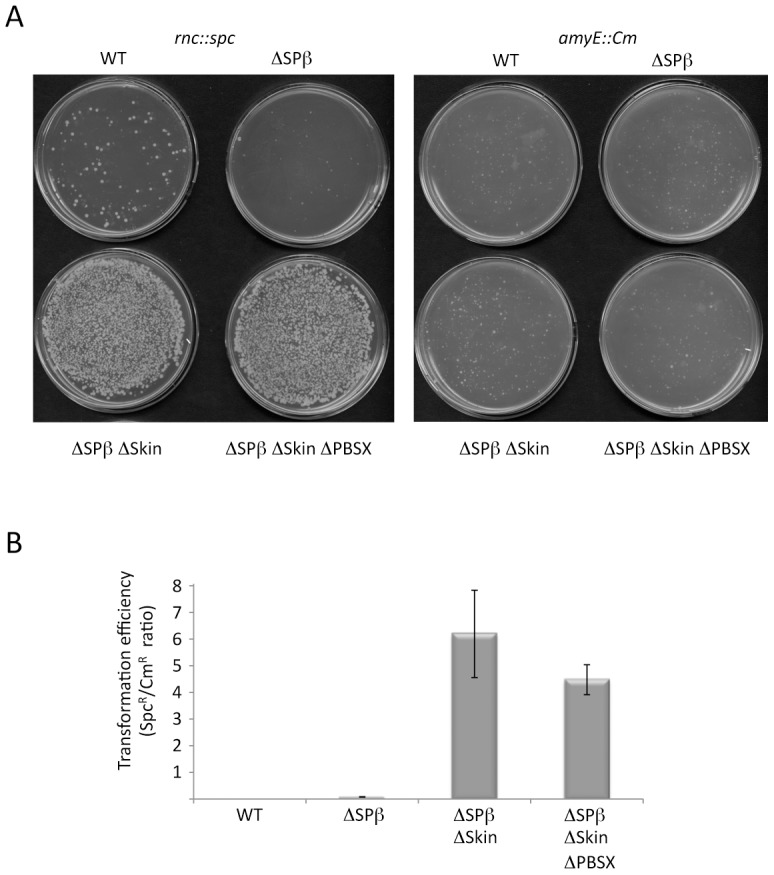
The *rnc* gene can be deleted in strains lacking Skin and SPβ prophages. (A) Agar plates showing colony growth after transformation of wild-type (WT) strains and strains lacking up to three prophages with 5 µg CCB302 (*rnc::spc amyE::Pxyl-rnc* Cm) chromosomal DNA, selected for spectinomycin (left panel) or chloramphenicol (right panel) resistance. (B) Histograms showing transformation efficiencies (number of Spc^R^ colonies/number Cm^R^ colonies) for the different prophage deficient strains.

*B. subtilis* strains lacking SPβ, Skin and a third prophage, PBSX, have been constructed previously [30]. We therefore asked whether it was possible to delete the *rnc* gene in strains lacking one (SPβ), two (SPβ and Skin) or all three of these prophages. Using chromosomal DNA from a *B. subtilis* strain (CCB 302), in which most of the *rnc* open-reading frame (ORF) has been replaced by an ORF encoding spectinomycin resistance (*rnc::spc*) and which survives through the presence of a xylose-dependent promoter expressing RNase III integrated at the *amyE* locus (*amyE::Pxyl-rnc* Cm^R^), we transformed each of the three prophage deficient strains and determined whether we could obtain spectinomycin resistant (Spc^R^) colonies. As a control for competence levels in each strain, we spread an aliquot of the transformation mixture on plates containing chloramphenicol (Cm) and counted the number of colonies having acquired the Cm-resistant construct at *amyE*. In wild-type cells or in cells lacking SPβ, very few Spc^R^ colonies were obtained, whereas in cells lacking both SPβ and Skin, or those lacking all three prophages, hundreds of colonies grew overnight (Figure 3A). When normalized for transformation efficiency (number of Spc^R^ colonies divided by number of Cm^R^ colonies), at least 100-fold more Spc^R^ colonies were obtained in strains lacking SPβ and Skin prophages compared to wild-type strains or strains lacking SPβ alone (Figure 3B). These results suggest it is possible to delete the RNase III gene from strains lacking SPβ and Skin.

### Natural suppressors of the *rnc::spc* mutation have excised Skin

**Figure 4:**
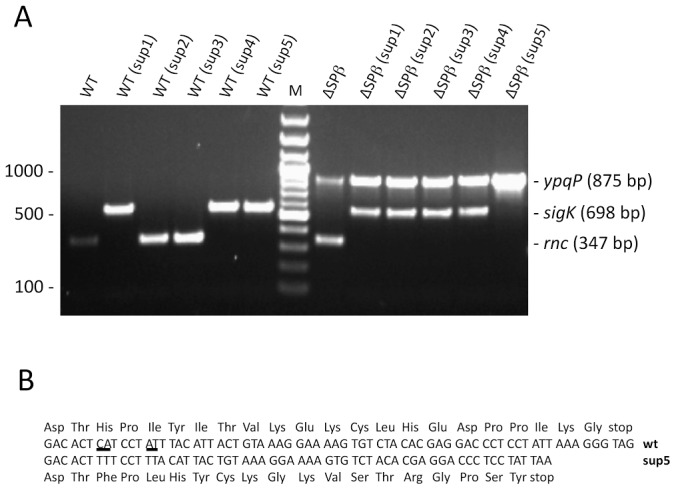
Suppressors of the *rnc::spc* mutation lack the Skin prophage. (A) Agarose gel showing multiplex PCR analysis of *rnc::spc* suppressors in wild-type (WT) and ΔSPβ strains. A PCR product corresponding to the reconstituted *ypqP* and *sigK* genes is indicative of excision of the SPβ and Skin prophages, respectively. Spontaneous Spc^R^ colonies show a PCR product for the *rnc* gene, while successfully deleted *rnc* strains do not give a corresponding PCR product. A DNA marker (bp) is shown in the lane labeled M. (B) Sequence comparison of the *txpA* gene in wild-type (wt) and the sup5 mutant shown in panel A.

We next asked what was the nature of the few Spc^R^ transformants obtained with the wild-type and SPβ-deficient strains. Given the result above, it was conceivable that suppressor strains might be obtained by excision of the prophages under selective pressure. We therefore analyzed some of the Spc^R^ transformants obtained in wild-type and SPβ-deficient strains from a number of different experiments to determine their genotypes. First, none of about 100 colonies tested (23 wild-type; 84 *Δ*SPβ) were Cm^R^, indicating that their viability was not due to simultaneous transfer of the *Pxyl-rnc* construct at *amyE*. We then examined the genotype of about 30 Spc^R^ transformants by multiplex colony PCR using oligonucleotide pairs specific for the *rnc*, *sigK* and *ypqP* genes. The Skin and SPβ prophages interrupt the *sigK* and *ypqP* genes, respectively, and thus spontaneous excision events can be observed by restoration of these genes and the generation of 567 and 880 bp PCR products. An intact *rnc* gene is indicated by a 347 bp PCR product. Three different classes of Spc^R^ transformants were obtained. A number of Spc^R^ transformants in wild-type strains retained an intact *rnc* gene (10 of 19 tested; see Figure 4A for examples), indicating a background level of spontaneous spectinomycin resistance in this strain. This was also evident from the growth of a significant number of Spc^R^ colonies in control transformations with water instead of CCB302 DNA (data not shown). All of the wild-type Spc^R^ transformants in which *rnc* had been successfully inactivated (9 of 19 tested) had also excised the Skin prophage (see Figure 4A for examples). It is therefore possible to obtain suppressors of the *rnc::spc* mutation by excising Skin. Curiously, none of the suppressors tested had excised SPβ, suggesting that of the two prophages, the presence of Skin is the most detrimental in an RNase III mutant strain. Over time, these suppressors show a pseudolysis phenotype on plates, however, suggesting all is not well in these strains. Almost all Spc^R^ transformants of the ΔSPβ strain had also excised Skin (13 of 14 tested; see Figure 4A for examples), confirming the idea that Skin is more deleterious than SPβ in strains lacking RNase III. The only suppressor that had not excised Skin (sup5) had a frame shift-mutation in the *txpA* gene (Figure 4B), suggesting that the TxpA peptide is the key Skin-encoded toxic moiety in the absence of RNase III.

### Expression of the *txpA* and *yonT* toxin mRNAs account for the lethality of the Skin and SPβ prophages in RNase III mutants

**Figure 5:**
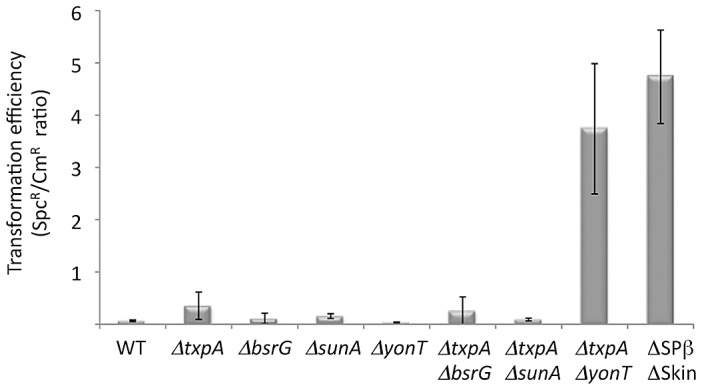
The *rnc* gene can be deleted in strains lacking TxpA and YonT toxins. Histogram showing transformation efficiencies (number of Spc^R^ colonies/number Cm^R^ colonies) for strains lacking different toxin genes or mRNAs.

To determine which prophage genes were responsible for the toxic effects in the absence of RNase III, we constructed strains no longer expressing the different RNase III-sensitive toxin mRNAs. Expression of the *txpA* gene was abolished by making a markerless deletion of its -10 promoter region (*txpA -10Δ*) using the pMAD system [31] and confirmed by Northern blot (data not shown), while the *bsrG*, *sunA* and *yonT* genes were inactivated by antibiotic resistance cassettes (Table S2). Transformation of the *txpA*, *sunA*, *bsrG* or *yonT* single mutants, with CCB302 chromosomal DNA had little effect on the number of Spc^R^ colonies or the Spc^R^/Cm^R^ ratio obtained overnight compared to wild-type (Figure 5). Based on the observation made above with the suppressor strain sup5, we assumed that TxpA was the relevant toxin in Skin and combined the *txpA -10Δ* mutation with that of each of the three toxin genes of SPβ. Only transformation of the *txpA -10Δ yonT* double mutant with CCB302 DNA yielded a similar Spc^R^/Cm^R^ colony ratio to strains lacking both Skin and SPβ prophages, suggesting these two toxin-encoding mRNAs are the primary determinants of cell toxicity in the absence of RNase III. To confirm this, we compared the growth rates of the *ΔSkin ΔSPβ Δrnc* and *txpA -10Δ ΔyonT Δrnc* strains with those of their parental strains lacking prophages or toxin genes. Deletion of the *rnc* gene in these two genetic contexts had no significant effect on doubling time in LB medium (Table 1), confirming that the major role of RNase III in *B. subtilis* under these growth conditions is to protect against the expression of these two foreign toxin-encoding mRNAs.

**Table 1:** Doubling time of *rnc* mutants and parental strains.

Strain	Doubling time (min)
WT (W168)	23.9+/−1.4
ΔSkin ΔSPβ	24.0+/−1.3
ΔSkin ΔSPβ *Δrnc*	24.9+/−1.8
*txpA -10Δ ΔyonT*	26.7+/−3.0
*txpA -10Δ ΔyonT Δrnc*	25.6+/−1.4

Values are the average of 3 independent experiments.

### The degradation pathway of RatA

We decided to further investigate the mechanism of RatA-induced degradation of *txpA* by RNase III, by studying the degradation pathways of the individual RNAs and the RatA/*txpA* complex. We were also curious about the sensitivity of the RatA antitoxin to the single-strand specific enzyme RNase Y, as we had expected that both toxin and antitoxin RNAs would be sensitive to RNase III.

**Figure 6:**
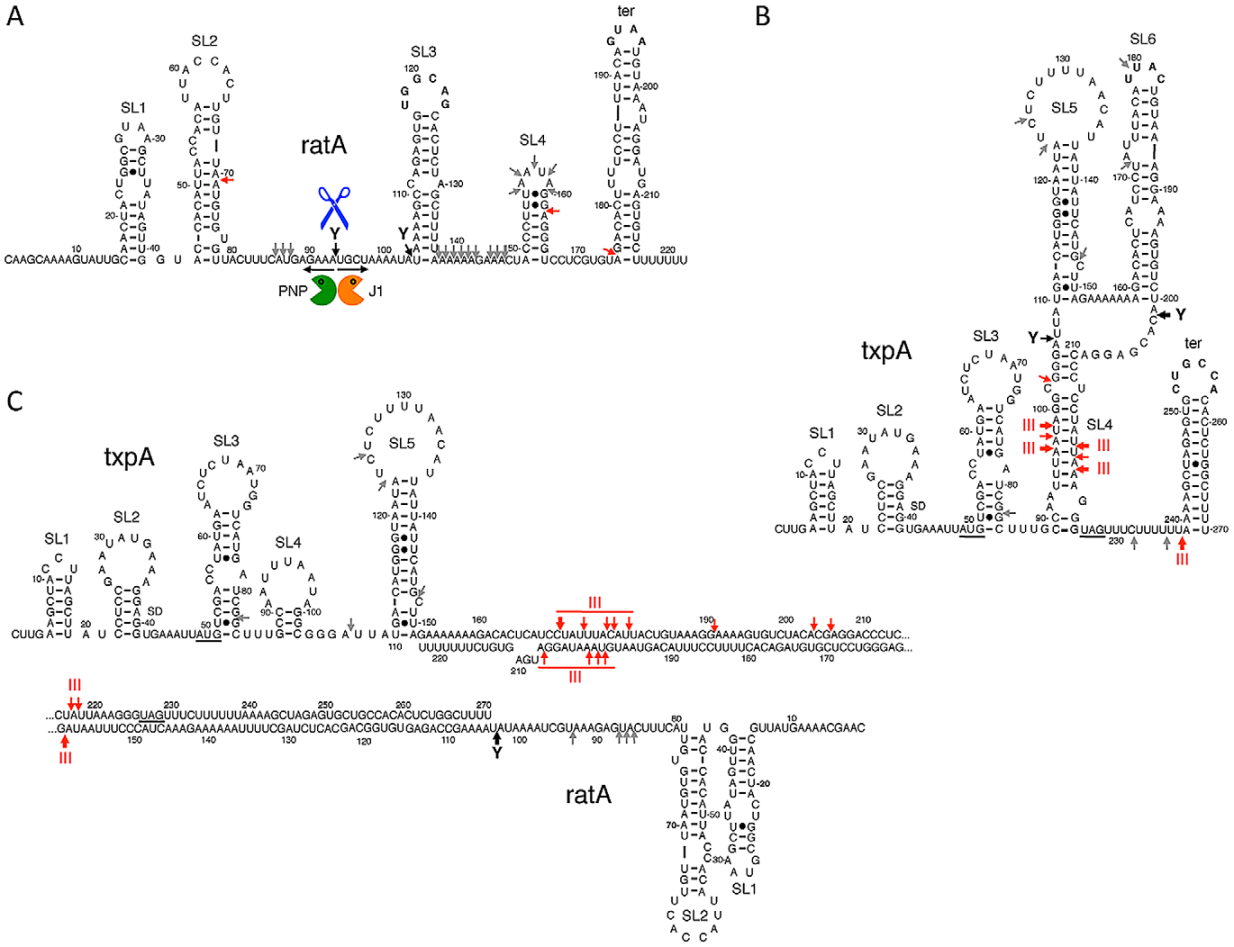
Secondary structures of RatA, *txpA*, and the RatA/*txpA* hybrid. Stem-loop (SL) structures and transcription terminators (ter) are labeled. Strong RNase III cleavages sites are shown as thick red arrows and minor sites as thin red arrows. Strong RNase Y cleavage sites are shown as black arrows and minor sites as grey arrows. The main degradation pathway *in vivo* is represented by RNase Y cleavage (blue scissors), followed by degradation of the upstream and downstream fragments by PNPase (green Pacman symbol) and RNase J1 (orange Pacman symbol), respectively. (A) Secondary structure of RatA. (B) Secondary structure of *txpA* (C) Secondary structure of RatA/*txpA* hybrid.

The stabilization of RatA under conditions of RNase Y depletion suggested that the initial endonucleolytic cleavage of this asRNA is performed by RNase Y. When RNase J1 was depleted, a number of 3′ proximal RatA degradation intermediates accumulated on high resolution gels, clustered around 130 nts and 75 nts in length (Figure S4A). This suggests that the major cleavage site by RNase Y and entry point for RNase J1 is around nt 90 of RatA, just upstream of the region of complementarity with *txpA*. The accumulation of the ∼75-nt species could either be due to the stabilization of the downstream product of a secondary endonucleolytic cleavage by an unknown enzyme or due to the incomplete depletion of RNase J1 and a stalling of the small amounts of remaining enzyme at the base of secondary structure. The 5′ ends of the ∼130-nt and ∼75-nt species were mapped by primer extension to nts 91 and to two clusters of bands around nts 97–99, and nts 145–147, respectively (Figure S5), both at the base of secondary structures (see below). In the absence of PNPase, the major 3′-5′ exoribonuclease in *B. subtilis*, an ∼80 nt 5′ proximal species accumulated (Figure S6), also consistent with an initial cleavage by RNase Y around nt 90 and a difficulty of the remaining 3′-5′ exoribonucleases in degrading past stem loop 2 (see below). A model for the degradation pathway of RatA is shown in Figure 6A, with cleavage by RNase Y around nt 90 giving access to both PNPase and RNase J1.

We next examined the degradation pattern of RatA in the absence of *txpA*, using the strain in which the -10 region of the *txpA* promoter was deleted. Surprisingly, the degradation pattern of RatA did not change; we observed the same stabilization of RatA in the absence of RNase Y and the accumulation of the same 3′ proximal degradation intermediates of around 130 nts and 75 nts in cells depleted for RNase J1 (Figure S4B). There were two possible explanations for this result; either the structure of RatA does not change significantly upon hybridization to *txpA*, or RatA is produced in large excess over *txpA*, in which case the degradation pathway observed in wild-type cells is primarily that of RatA alone, rather than the RatA/*txpA* hybrid. Quantitative Northern blotting using known quantities of *in vitro* transcribed RatA and *txpA*, showed that RatA is present in about 15-fold excess over *txpA* in wild-type cells (Figure S7), in favor of the latter hypothesis.

### Degradation of *txpA* is dependent on both RatA and RNase III

**Figure 7:**
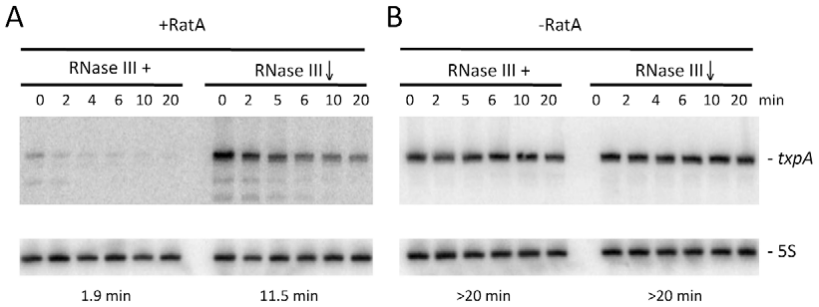
Degradation of *txpA* (AUG→AAG) mRNA by RNase III is RatA-dependent. Northern blots performed on RNA isolated at times (min) after rifampicin addition in (A) strain CCB467 *txpA* (AUG→AAG) and (B) strain CCB468 *txpA* (AUG→AAG) P*ratA::ery*, depleted or not for RNase III. Northerns were re-probed for 5S rRNA (5S) for normalization. Half-lives are given below each panel.

In wild-type cells, turnover of *txpA* is primarily dependent on RNase III. The fact that RatA is present in large excess over *txpA* in this strain suggests that, in the case of *txpA*, we primarily measured an effect of enzyme depletion on the RatA/*txpA* hybrid. We failed in attempts to make a deletion of the -10 region of the RatA promoter to study the degradation of wild-type *txpA* in the absence of RatA *in vivo*, presumably because of TxpA toxicity. To circumvent this problem, we made an untranslated derivative of the *txpA* mRNA by changing its start codon to AAG. This mutation falls outside of the region of RatA/*txpA* complementarity and is not anticipated to affect the secondary structure of *txpA* (see below) or the ability of these two RNAs to form a duplex. Like the wild-type *txpA* mRNA, the AUG→AAG mRNA was stabilized in strains depleted for RNase III (Figure 7), showing that the degradation of this non-translated derivative is still RNase III dependent. The difference in half-life compared to that measured with the wild-type *txpA* in Figure 1 (11.5 mins vs. >20 mins) is most likely explained by different RNase III depletion efficiencies in the two experiments.

We were able to successfully replace the *ratA* promoter region with an antibiotic resistance cassette in the context of the *txpA* (AUG→AAG) mutation, showing that the peptide rather than the mRNA is toxic in *B. subtilis*, and allowing us to study the *txpA* mRNA independently of RatA. In the absence of RatA, the *txpA* (AUG→AAG) mRNA was highly stabilized, regardless of whether or not RNase III was present. These experiments show that the rapid turnover of the *txpA* mRNA is dependent on both RatA and RNase III; in the absence of either one, the *txpA* mRNA is extremely stable.

### *txpA* and RatA form an extended hybrid that is a substrate for RNase III cleavage

To study the degradation mechanisms of RatA and *txpA* further, we turned to *in vitro* experiments. We made 5′ labeled RatA and *txpA* RNAs *in vitro* using T7 RNA polymerase and first probed the secondary structures of the individual RNAs and the RatA/*txpA* hybrid using the single-stranded endonuclease activity of RNase J1. We have previously shown that this property of RNase J1 can be exploited to determine both known and unknown RNA folding patterns [32]. The structure probing data are shown in Figures S8, S9, and S10 and the predicted secondary structures of the individual RatA and *txpA* RNAs and the RatA/*txpA* hybrid are shown in Figure 6. The RatA asRNA forms four stem-loop structures (labeled SL1–4) in addition to the transcription terminator (ter). The *txpA* RNA forms up to six helical structures (SL1–6) in addition to the terminator *in vitro*. The significantly reduced intensity of RNase J1 cleavages between nts 137 and 169 of RatA in the RatA/*txpA* hybrid (Figure S8) is consistent with an extended duplex comprising most of the last 120 nts of each RNA. The duplex does not appear to extend all the way to the 3′ end however, as an RNase J1 hypersensitive region appears in nts corresponding to the downstream strand of the RatA terminator sequence (marked with an asterisk in Figure S8B) when RatA is complexed with *txpA*.

**Figure 8:**
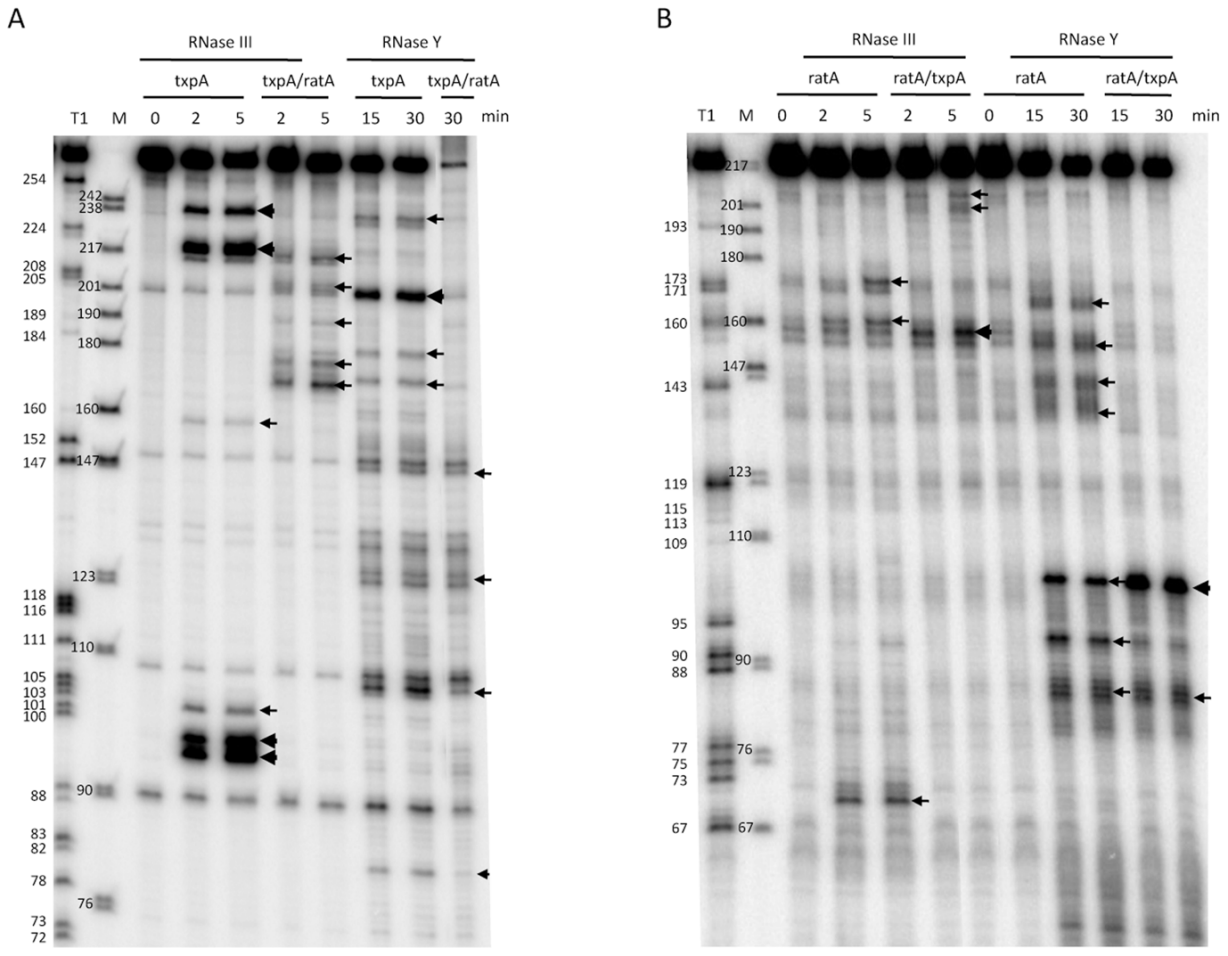
Cleavage of RatA, *txpA*, and the RatA/*txpA* hybrid by RNase III and RNase Y. (A) Cleavage of 5′ labeled *txpA* (0.5 pmol) alone or hybridized to a two-fold excess of unlabeled RatA by RNase III (3 ng) and RNase Y (2 µg) for the times indicated. A size standard is shown in lane M and an RNase T1 digestion of 5′ labeled *txpA* is shown in the lane labeled T1. (B) Cleavage of 5′ labeled RatA alone or hybridized to a two-fold excess of unlabeled *txpA* by RNase III and RNase Y for the times indicated. A size standard is shown in lane M and an RNase T1 digestion of 5′ labeled RatA is shown in the lane labeled T1.

Incubation of the *txpA* mRNA with purified RNase III revealed a major cleavage site at nts 96–98 and a corresponding cleavage site at nts 218–220, consistent with cleavage on both sides of the helical structure SL4 by RNase III (Figure 8A). When *txpA* was hybridized to RatA, these cleavages disappeared completely and different set of RNase III cleavage sites was seen between nts 170–179 of *txpA*. Thus, binding of RatA changes the structure of *txpA*, creating new RNase III-sensitive sites. Corresponding cleavages were seen between nts 198–206 of the RatA RNA (Figure 8B), suggesting that both sense and antisense partners are simultaneously cleaved by RNase III. A second pair of RNase III cleavage sites was observed at nts 217/218 of *txpA* and nt 159 of RatA.

Since the RatA asRNA is a substrate for RNase Y *in vivo*, we also subjected both RNAs to RNase Y cleavage *in vitro*. Incubation of the RatA mRNA with purified RNase Y, revealed a number of minor sites of endonucleolytic cleavage, indicative of a relatively relaxed specificity for this enzyme *in vitro* (Figure 8B). The site most relevant to the pathway seen *in vivo* is likely to be that at nt 93, close to the major site of RNase J1 access to the RatA RNA *in vivo* (nt 91). Upon hybridization to *txpA*, the *in vitro* cleavage by RNase Y at nt 93 was considerably weaker while that at nt 103, immediately upstream of the duplex, was significantly enhanced. Furthermore, the clusters of cleavages from nt 137 to 159 were lost, consistent with the creation of an extended RatA/*txpA* duplex in this region. Although a relatively prominent RNase Y cleavage site was observed at nt 201 of the *txpA* mRNA alone (Figure 8A), this site is in the region of complementarity to RatA and is not likely to exist very much *in vivo* under conditions of a large excess of RatA. Indeed, cleavage at this site no longer occurs when *txpA* is hybridized to RatA *in vitro*.

## Discussion

In this paper we have determined that the essential function for RNase III in *B. subtilis* is to protect it from the expression of toxin genes borne by the Skin and SPβ prophages, via antisense RNA. The cleavage of double-stranded RNA by RNase III-related enzymes as part of host defense mechanisms against virus infection is well-documented. The role played by Dicer in the generation of siRNAs in the process of RNA interference, for example, is a fundamental component of this innate viral defense system (for recent review, see [33]). A recent study has also shown that the CRISPR system, initially characterized as a self-contained defense mechanism against prokaryotic plasmids and phages, uses the host enzyme RNase III to help generate the short protective crRNAs in some bacteria [6]. Although *B. subtilis* does not contain a CRISPR cassette, our data shows that it nonetheless relies on RNase III to protect it from prophage gene expression, through antisense RNA.

The mode of action of the TxpA and YonT toxins is not known. Both encode short peptides of 59 and 58 amino acids, respectively, with little sequence homology between them. Both have hydrophobic N-termini, predicted to form a transmembrane domain [27], and hydrophilic C-termini; the last 20 amino acids of YonT are very highly positively charged (75% arginine or lysine residues). TxpA has been shown to cause cell-lysis in *B. subtilis* [24] and YonT to cause growth arrest upon induction in *E. coli* [27]. We had previously noticed that TxpA (previously known as YqdB) and YonT have the two strongest Shine-Dalgarno (SD) sequences in *B. subtilis* [34], with 11–12 possible base-pairs with the anti-SD 3′ end of 16S rRNA. It is not known what effect this has on toxin translation levels; while a very strong SD should help ribosome recruitment, these ribosomes may have difficulty escaping the SD. The observation that suppressor colonies, obtained upon transformation of either wild-type or ΔSPβ cells with the *rnc::spc* construct, have excised the Skin prophage or mutated TxpA, suggests that TxpA is the more toxic of the two peptides (it also has the stronger match to the anti-SD in 16S rRNA).

Paradoxically, we were able to obtain suppressor strains lacking only the Skin prophage, but for optimal transformation efficiency with the *rnc::spc* construction, it was necessary to remove both Skin and SPβ. Similarly, one of the suppressor strains had a frame-shift mutation in *txpA*, yet the transformation efficiency of the *txpA -10Δ* mutant was much lower than that of the *txpA yonT* double mutant. We cannot rule out the possibility that the suppressors have additional mutations that affect the levels or expression of SPβ genes (see below). However, it is also important to consider that the pressure on the cell to produce colonies is not the same in both cases. In the transformation efficiency assay, cells are additionally being asked to go through the process of becoming competent, a complex developmental program put in place by starving cells. Although the specific effect of competence on toxin gene expression is not known, expression of *yonT* has recently been shown to reach a peak about 30 mins after glucose starvation [35] and this could have a significant effect on the number of colonies recovered in the transformation assay. The fact that the *Δskin SPβ^+^* suppressors of the *Δrnc::spc* mutation show a pseudolysis phenomenon on plates suggests that they are not completely healthy, consistent with the idea that the lack of both prophages and their encoded toxins is the optimal configuration for growth and survival in the absence of RNase III.

The Bechhofer laboratory has previously isolated two suppressor strains (BG322 and BG323) in which the *rnc* gene was successfully inactivated [13]. We also examined both of these strains for the presence of the Skin and SPβ prophages. Both BG322 and BG323 have excised Skin, but appear to have different propensities to excise SPβ during growth (Figure S11). BG323 consistently has a greater proportion of cells lacking SPβ than BG322, but only a minor fraction of cells in freshly plated colonies of either strain have lost SPβ (data not shown), suggesting that this prophage is not stably cured and that these strains have additional mutations that influence their ability to excise SPβ.

We performed *in vivo* and *in vitro* experiments to probe the mechanism of RatA-mediated destabilization of *txpA* mRNA catalyzed by RNase III. We showed that degradation of a non-translated derivative of *txpA* (AUG→AAG) is dependent on both RatA and RNase III *in vivo* and that hybridization of the *txpA* and RatA RNAs generates a highly sensitive substrate for RNase III *in vitro*. We also determined the secondary structures of the two individual RNAs and the RatA/*txpA* hybrid. The apical loops of SL3 and the transcription terminator of RatA are complementary to those of the terminator of *txpA* and SL6, respectively (Figure 6). The formation of sense/antisense RNA complexes is often initiated through loop-loop or ‘kissing’ interactions [36], [37] and RatA and *txpA* can potentially use the same mechanism to initiate hybrid formation. The extent to which the duplex extends in either direction from the initial interaction site depends on the RNAs in question. For the copA/copT sense/antisense pair, involved in copy number control of the plasmid R1, the duplex does not extend very far from the initial interaction site before getting trapped in a four-way junction with side-by-side helices [37]. The RatA/*txpA* duplex, on the other hand, seems to extend over most of the 120 nucleotides of complementarity between the two RNAs.

The formation of an extended duplex between RatA and *txpA* generates a substrate for RNase III and this is the primary mechanism of *txpA* control *in vivo*. Indeed, we can calculate that RatA remains in about a 3.5 fold excess over *txpA* in strains depleted for RNase III; despite this continued excess of RatA, the increased levels of the *txpA* mRNA are toxic to *B. subtilis*, presumably because the *txpA* mRNA can still be translated when paired to RatA. The control of *bsrG* by *as-bsrG(SR4)* also occurs at the level of RNA turnover, mediated by RNase III [28], while the control of the type I TA system of *Enterococcus faecalis*, Fst, occurs primarily at the translational level [38]. The products of *txpA* cleavage by RNase III appear to be degraded primarily by PNPase, judging from the accumulation of many 5′ proximal *txpA* fragments in a *pnp* mutant strain *in vivo* (Figure S6B). The *txpA* mRNA is also a good substrate for RNase III *in vitro* even in the absence of RatA, through the formation of the double-stranded helix SL4 (Figure 6). SL4 can no longer form when *txpA* is hybridized to RatA. *In vivo*, where there is a large excess of RatA, it is therefore unlikely that this structure forms very often. Even in the absence of RatA, the *txpA* (AUG→AAG) mRNA is stable in cells containing RNase III (Figure 7), suggesting that this potential RNase III cleavage site in *txpA* is also unlikely to be accessible when such conditions arise *in vivo*.

Depletion of RNase III also leads to an accumulation of the *yonT* mRNA and a longer species that encodes both YonT and YoyJ. A ∼100-nt asRNA to *yonT* was detected previously by Northern blot [27] and we have seen this species is stable even in the presence of RNase III (Figure 2C). A recent tiling array analysis suggests this asRNA also overlaps the beginning of the YoyJ reading frame (Figure 2A) and may therefore control the expression of both genes. YoyJ encodes an 83 amino acid protein of unknown function.

It is not clear what the role TxpA or YonT play in the biology of the prophage. They may simply be part of a prophage maintenance system, by killing *B. subtilis* cells that do not constantly synthesize their asRNAs. However, they could also play a role in linking prophage biology to the physiology of the cell. It has recently been shown, for example, that levels of the *bsrG* toxin mRNA of SPβ are decreased about 10-fold at 48°C [28]. Interestingly, both *txpA* and *yonT* show high expression levels under conditions of glucose exhaustion [35]. TxpA expression is additionally sensitive to both high and low phosphate concentrations, while *yonT* is induced in the presence of mitomycin C, which induces the SOS response. It will be interesting to determine whether these conditions affect either the behavior of the prophage or the host-cell in a toxin-dependent manner. In a recent study, depletion of the Skin transcriptional repressor (SknR) was shown to cause cell death through overexpression of two proteins YqaM and YqaH, which bind to DnaA and DnaC, respectively [39]. The Skin prophage appears thus to have a variety of options that allow it to slow or halt the growth of its host cell.

## Materials and Methods

### Construction of bacterial strains

The *B. subtilis* strains used in this study were derivatives of W168 or 168 *trp* ^−^. Strains lacking SPβ, Skin and PBSX prophages (168 *trp^−^* background) have been described previously [30] and were a kind gift from J.M. van Dijl. A Skin-less derivative of W168 was kindly constructed by P. Stragier and named CCB297.

Strain CCB302 (*rnc::spc amyE*::pX-*rnc* Cm) was constructed as follows. The *rnc* gene was amplified from *B. subtilis* chromosomal DNA using oligos CC816/817 (Table S1), digested with SpeI and BamHI and cloned in pX [40] cleaved with the same enzymes. The resulting plasmid, pX-*rnc*, was integrated in the *amyE* locus of Skin-less strain CCB297 to create CCB298. The *rnc* gene of CCB298 was then interrupted by a spectinomycin resistance cassette using chromosomal DNA from strain BG324 [13] to create strain CCB302. Growth of this strain is xylose-dependent.

Strains CCB034 (*rnjA*:pMUTIN-*rnjA*), CCB288 (*rnc::spc amyE::Pspac-rnc* Cm) and CCB294 (*rny::spc amyE::Pspac-rny* Cm) have been described previously [20], [23]. RNase depletions were performed as in [20], [23].

Strain CCB363 (SPβ::PIID-*sspB* kan) was constructed by transforming W168 with chromosomal DNA from MO4738 SPβ::PIID-*sspB* kan *spoIIIE::Tc*, a kind gift from P. Stragier. This chromosomal DNA was also used to transform CCB297 to create CCB364 ΔSkin SPβ::PIID-*sspB* kan.

Strain CCB325 (*txpA -10Δ*) was made by markerless mutation of the -10 promoter region of *txpA* on the W168 chromosome using plasmid pMAD-I according to [31]. Plasmid pMAD-I was constructed as follows. Overlapping upstream and downstream fragments containing the *txpA* -10 promoter deletion were amplified using oligo pairs CC907/908 and CC909/910, respectively. The overlapping fragments were then assembled in a new PCR reaction with CC907 and CC910, digested with BamHI and cloned in pMAD [31], a kind gift from M. Débarbouillé.

Strain CCB361 *bsrG::kan* was constructed by building a PCR fragment containing upstream and downstream regions of the *bsrG* gene flanking a kanamycin resistance cassette by over lapping PCR, using oligos CC1015–1020 (Table S1), and transforming in W168. Chromosomal DNA from this strain was used to transform strain CCB325 to create CCB368 *txpA -10Δ bsrG::kan*.

Strain CCB377 *sunA::kan* was made by transforming W168 by chromosomal DNA from a *sunA::kan* strain, kindly provided by J.M. van Dijl. This chromosomal DNA was also transformed into CCB325 to create CCB402 *txpA -10Δ sunA::kan*.

Strain CCB413 *yonT::ery* was constructed by building a PCR fragment containing upstream and downstream regions of the *yonT* gene, flanking an erythromycin resistance cassette using oligos CC1071–CC1077 (Table S1) and transforming in W168. Chromosomal DNA from this strain was used to transform strain CCB325 to create CCB414 *txpA -10Δ yonT::ery*.

Strain CCE192, used to overexpress N-terminal His tagged *B. subtilis* RNase Y, was constructed as follows. The RNase Y gene (*ymdA/rny*) lacking sequences corresponding to the N-terminal transmembrane domain was amplified by PCR using oligos CC657 and CC658 (Table S1), cleaved by NdeI and BamHI and cloned in pET28a (Novagen) cut with the same enzymes. The resulting plasmid pET28-ΔTM-YmdA was transformed into *E. coli* strain BL21 CodonPlus cells to yield strain CCE192.

Strain CCB456 was made by markerless mutation of the *txpA* start codon AUG to AAG using plasmid pMAD-III according to [31]. Plasmid pMAD-III was constructed as follows. Overlapping upstream and downstream fragments containing the AAG mutation were amplified using oligo pairs CC907/1129 and CC1130/910, respectively. The overlapping fragments were then assembled in a new PCR reaction with CC907/910, digested with BamHI and cloned in pMAD.

Strain CCB461 was made by deleting the *ratA* promoter region (68 nts) with an erythromycin resistance cassette in strain CCB456. Upstream and downstream homology regions were assembled on either side of the antibiotic cassette by overlapping PCR using the following oligo pairs CC1151/1148, CC1147/1149 and CC1150/1152. The 3 overlapping fragments were then assembled in a new PCR reaction amplified by oligos CC1151 and CC1152 and used to transform CCB456.

Strains CCB467 and CCB468 were made by successively transferring the *amyE::Pspac-rnc* Cm, *rnc::spc* constructs and plasmid pMAP65 to strain CCB456 and CCB461, respectively.

### Northern blots and primer extension assays

Northern blots and primer extension assays were performed as described previously [20], [23].

### Multiplex PCR reactions

Multiplex PCR reactions were performed in the presence of the 6 oligonucleotides CC435/1011 (*rnc*), CC986/987 (*sigK*), 990/991 (*ypqP*) for 25 cycles (94°C for 30 sec, 53° for 30 sec, 72°C for 1 min).

### Structure probing experiments

Structure probing experiments using C-terminal His tagged RNase J1 (0.6 µg per reaction) have been described in [32]. The *txpA* and RatA transcripts were transcribed *in vitro* by T7 RNA polymerase (Ambion) using PCR templates with integrated T7 promoters (oligo pairs CC998/999 and CC1000/1001, respectively). RNAs were synthesized with a 5′OH group (using an 6-fold excess of guanosine over GTP) to facilitate 5′ labeling. RNAs were 5′ labeled using T4 polynucleotide kinase (Biolabs).

### *In vitro* RNase cleavage assays

The purification and assay of RNase III has been described previously [21]. N-terminal His-tagged RNase Y was purified from strain CCE192 on a Ni-NTA column as described previously [41] and dialyzed against elution buffer without imidazole. The final enzyme concentration was 4 mg/ml. RNase Y was assayed *in vitro* in 20 mM Tris pH 8.0, 8 mM MgCl_2_, 100 mM NH_4_Cl, 0.1 mM DTT.

## Supporting Information

Figure S1 The as-bsrH RNA shows similar RNase sensitivity to RatA, but the *bsrH* mRNA is insensitive to RNase III depletion. (A) Chromosomal context of the *bsrH/as-bsrH* toxin/antitoxin cassette present in the Skin prophage. (B) and (C) Northern blots performed on RNAs isolated at times (min) after rifampicin addition (150 µg/ml) in strains depleted for RNase III (CCB288), RNase Y (CCB294) and RNase J1 (CCB034), probed for *bsrH* and as-bsrH, respectively. Northerns were re-probed for 5S rRNA (5S) for normalization. Half-lives are given below each panel. The band labeled D in panel C (RNase J1) is a degradation intermediate of as-bsrH.
(TIF)

Figure S2 The *bsrG* and as-brsG (SR4) RNAs are stabilized in strains depleted for RNase III and RNase Y, respectively. (A) Chromosomal context of the *bsrG/as-bsrG* toxin/antitoxin cassette present in the SPβ prophage. (B) and (C) Northern blots performed on RNAs isolated at times (min) after rifampicin addition (150 µg/ml) in strains depleted for RNase III (CCB288), RNase Y (CCB294) and RNase J1 (CCB034), probed for *bsrG* and as-bsrG, respectively. Northerns were re-probed for 5S rRNA (5S) for normalization. Half-lives are given below each panel.
(TIF)

Figure S3 The *sunA* mRNA is overexpressed in a strain depleted for RNase III. (A) Chromosomal context of the *sunI-sunA* locus present in the SPβ prophage. (B) Northern blots performed on RNA isolated at times (min) after rifampicin addition in strains depleted for RNase III (CCB288). The Northern was re-probed for 5S rRNA (5S) for normalization. Half-lives are given below each panel.
(TIF)

Figure S4 The degradation profile of RatA is identical in wild-type strains and in strains no longer expressing *txpA*. (A) High resolution (5% polyacrylamide) Northern blots performed on RNAs isolated from wild-type cells at times (min) after rifampicin addition (150 µg/ml) in strains depleted for RNase III (CCB288), RNase Y (CCB294) and RNase J1 (CCB034). Migration positions (in nts) of an RNA marker are given to the right of the figure. (B) High resolution Northern blots performed on RNAs isolated from *txpA -10*Δ cells (CCB325) and *txpA -10*Δ cells depleted for RNase III (CCB348), RNase Y (CCB338) and RNase J1 (CCB337).
(TIF)

Figure S5 Mapping of 5′ ends of RatA intermediates that accumulate in strains depleted for RNase J1. Primer extension assay using oligo CC758 (Table S1) on 15 µg of total RNA isolated from wild-type strains (WT) and strain CCB034 (Table S2) grown in the presence and absence of IPTG. A sequence reaction performed with the same oligo on a PCR template of the *txpA*/RatA region (oligos CC795/796; Table S1) is shown to the left. The sequence is labeled as its reverse complement to facilitate direct reading.
(TIF)

Figure S6 RatA and *txpA* degradation intermediates accumulate in the absence of PNPase. Northern blots of RNA isolated from wild-type (WT), PNPase (SSB1030), RNase R (CCB021) and RNase PH (CCB308) mutants (Table S2) probed with (A) oligo CC862 (Table S1) specific for the 5′ end of RatA and (B) oligo CC861 (Table S1) specific for the 5′ end of *txpA*.
(TIF)

Figure S7 RatA is present in excess over *txpA* in wild-type cells. Quantitative Northern blot loaded with known quantities (in pg) of *in vitro* transcribed *txpA* and RatA RNAs, and either 5 or 15 µg of total RNA isolated from wild-type cells.
(TIF)

Figure S8 Structure probing of RatA RNA and RatA/*txpA* hybrid. *In vitro* transcribed 5′-labeled RatA RNA (0.5 pmol) alone hybridized to a 2-fold excess of unlabeled *txpA* were incubated with 0.6 µg RNase J1 for 2 or 5 minutes and loaded on a 5% polyacrylamide/urea gel. The RatA RNA was also digested with RNase T1 (Ambion) under denaturing conditions at the dilutions shown to reveal migration positions of G residues. A DNA size standard (in nts) is shown in the lane labeled M. (A) short migration (B) long migration with same samples.
(TIF)

Figure S9 Structure probing of *txpA* RNA and *txpA*/RatA hybrid. *In vitro* transcribed and 5′ -labeled *txpA* RNA (0.5 pmol) alone or hybridized to a 2-fold excess of unlabeled RatA were incubated with 0.6 µg RNase J1 for 2 or 5 minutes and loaded on a 5% polyacrylamide/urea gel. The 5′ -labeled *txpA* RNA was also digested with RNase T1 (Ambion) under denaturing conditions at the dilutions shown to reveal migration positions of G residues. A DNA size standard (in nts) is shown to the right. (A) short migration (B) long migration with same samples.
(TIF)

Figure S10 Summary of structure probing data for RatA, *txpA* and *txpA*/RatA hybrids. Mapped RNase J1 cleavages (arrowheads) on the best-fitting secondary structures of (A) RatA (B) *txpA* and (c) the *txpA*/RatA hybrid. Overlapping sequences of RatA and *txpA* are shown in red and green, respectively. The Shine-Dalgarno (SD) sequence, initiation and termination codons of *txpA* are shown in blue.
(TIF)

Figure S11 Suppressor strains BG322 and BG323 have excised the Skin prophage and have excised SPβ to different degrees. Agarose gel showing multiplex PCR analysis of *rnc::spc* suppressor strains. A PCR product corresponding to the reconstituted *ypqP* and *sigK* genes is indicative of excision of the SPβ and Skin prophages, respectively. Strains with a wild-type *rnc* gene give a 347 nt PCR fragment, while successfully deleted *rnc* strains do not give a PCR product. A DNA marker (bp) is shown in the lane labeled M.
(TIF)

Table S1 Oligonucleotides used in this study. Non-hybridizing sequences are in lower case letters.
(DOC)

Table S2 *B. subtilis* strains used in this study.
(DOC)
