# Supplementary figures and images for: The Essential Function of B. subtilis RNase III Is to Silence Foreign Toxin Genes
Source: PLoS Genet. 2012 Dec 27;8(12):e1003181. doi: 10.1371/journal.pgen.1003181 (PMC3531473; doi:10.1371/journal.pgen.1003181)

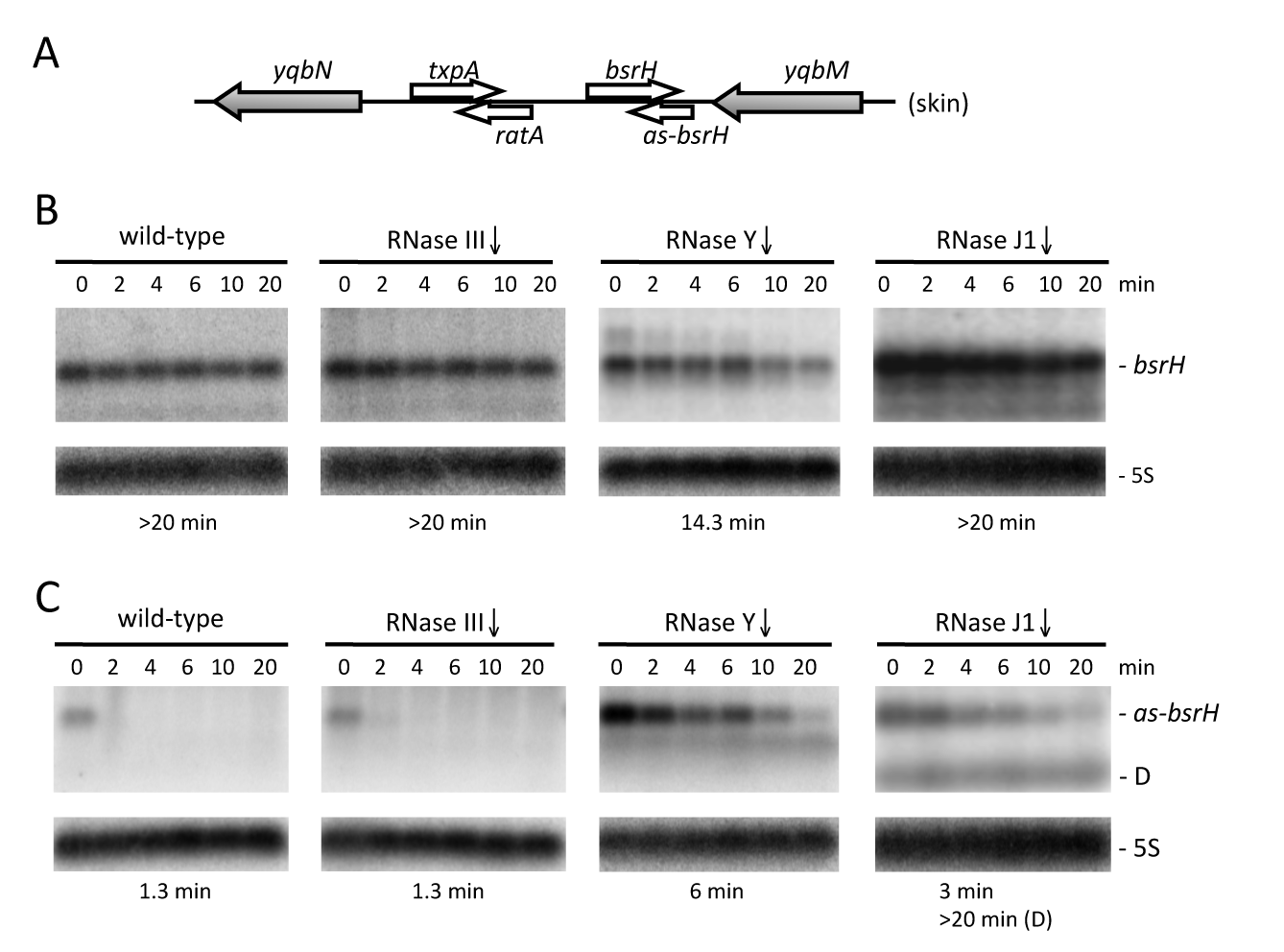

Supplement: Figure S1 — The as-bsrH RNA shows similar RNase sensitivity to RatA, but the bsrH mRNA is insensitive to RNase III depletion. (A) Chromosomal context of the bsrH/as-bsrH toxin/antitoxin cassette present in the Skin prophage. (B) and (C) Northern blots performed on RNAs isolated at times (min) after rifampicin addition (150 µg/ml) in strains depleted for RNase III (CCB288), RNase Y (CCB294) and RNase J1 (CCB034), probed for bsrH and as-bsrH, respectively. Northerns were re-probed for 5S rRNA (5S) for normalization. Half-lives are given below each panel. The band labeled D in panel C (RNase J1) is a degradation intermediate of as-bsrH. (TIF) [file pgen.1003181.s001.tif]

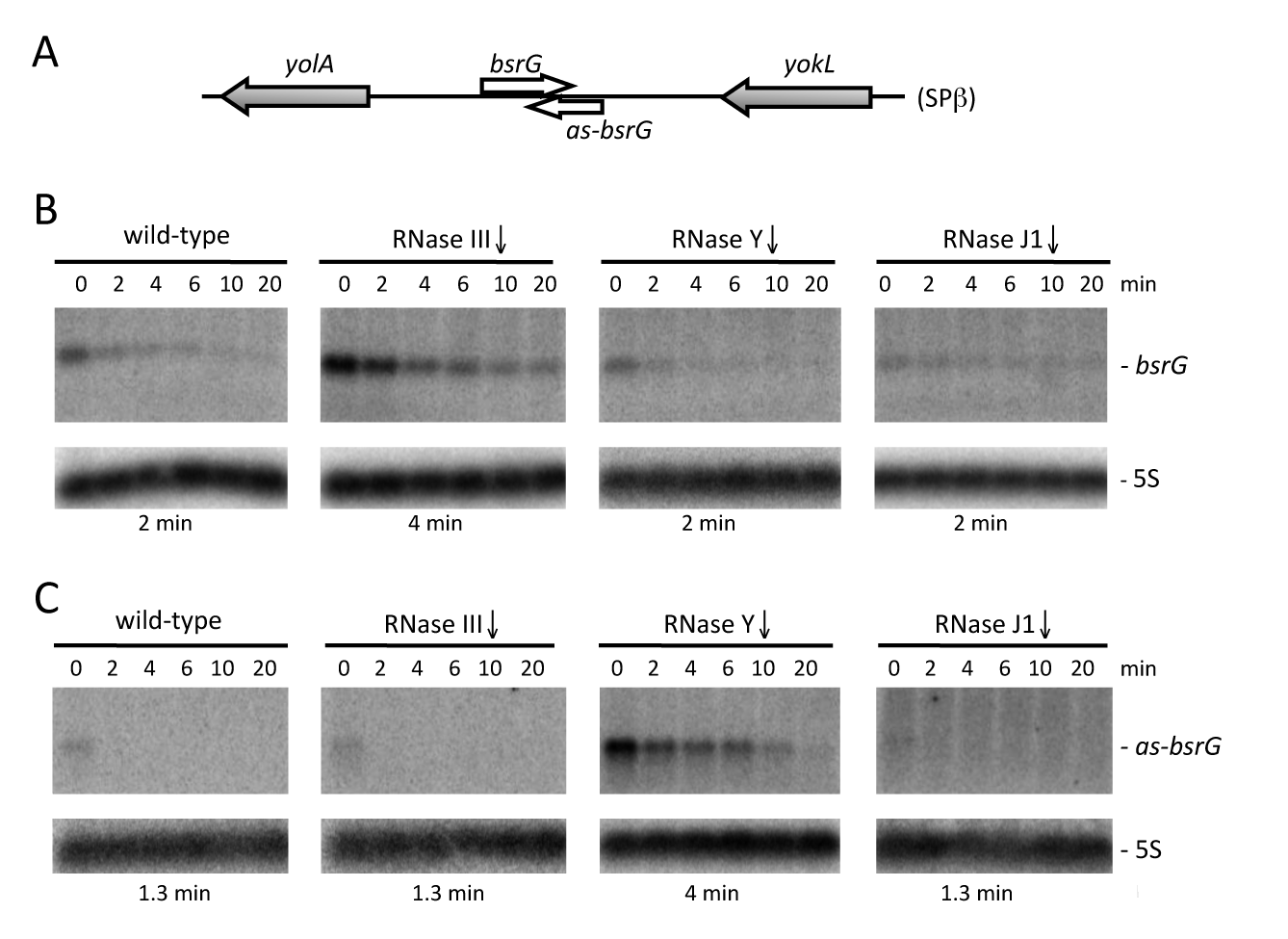

Supplement: Figure S2 — The bsrG and as-brsG (SR4) RNAs are stabilized in strains depleted for RNase III and RNase Y, respectively. (A) Chromosomal context of the bsrG/as-bsrG toxin/antitoxin cassette present in the SPβ prophage. (B) and (C) Northern blots performed on RNAs isolated at times (min) after rifampicin addition (150 µg/ml) in strains depleted for RNase III (CCB288), RNase Y (CCB294) and RNase J1 (CCB034), probed for bsrG and as-bsrG, respectively. Northerns were re-probed for 5S rRNA (5S) for normalization. Half-lives are given below each panel. (TIF) [file pgen.1003181.s002.tif]

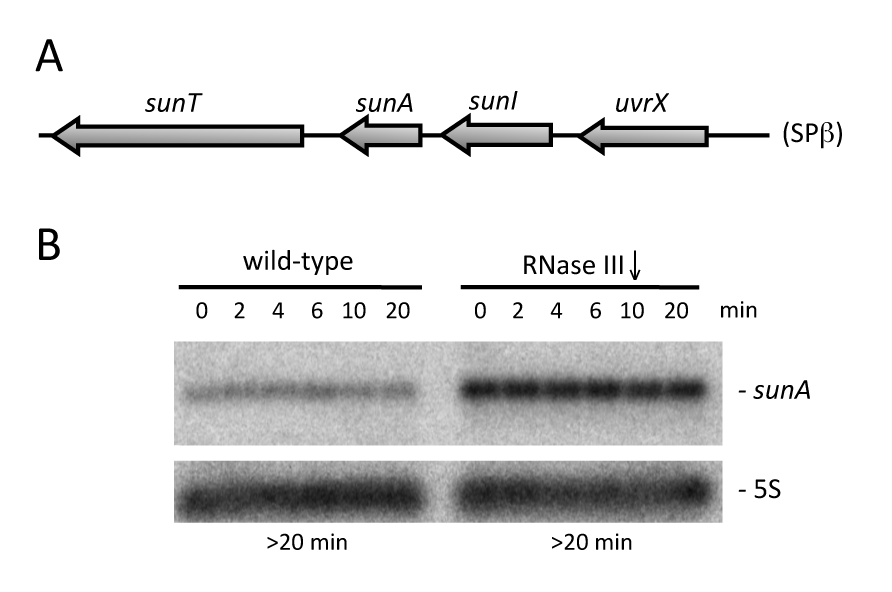

Supplement: Figure S3 — The sunA mRNA is overexpressed in a strain depleted for RNase III. (A) Chromosomal context of the sunI-sunA locus present in the SPβ prophage. (B) Northern blots performed on RNA isolated at times (min) after rifampicin addition in strains depleted for RNase III (CCB288). The Northern was re-probed for 5S rRNA (5S) for normalization. Half-lives are given below each panel. (TIF) [file pgen.1003181.s003.tif]

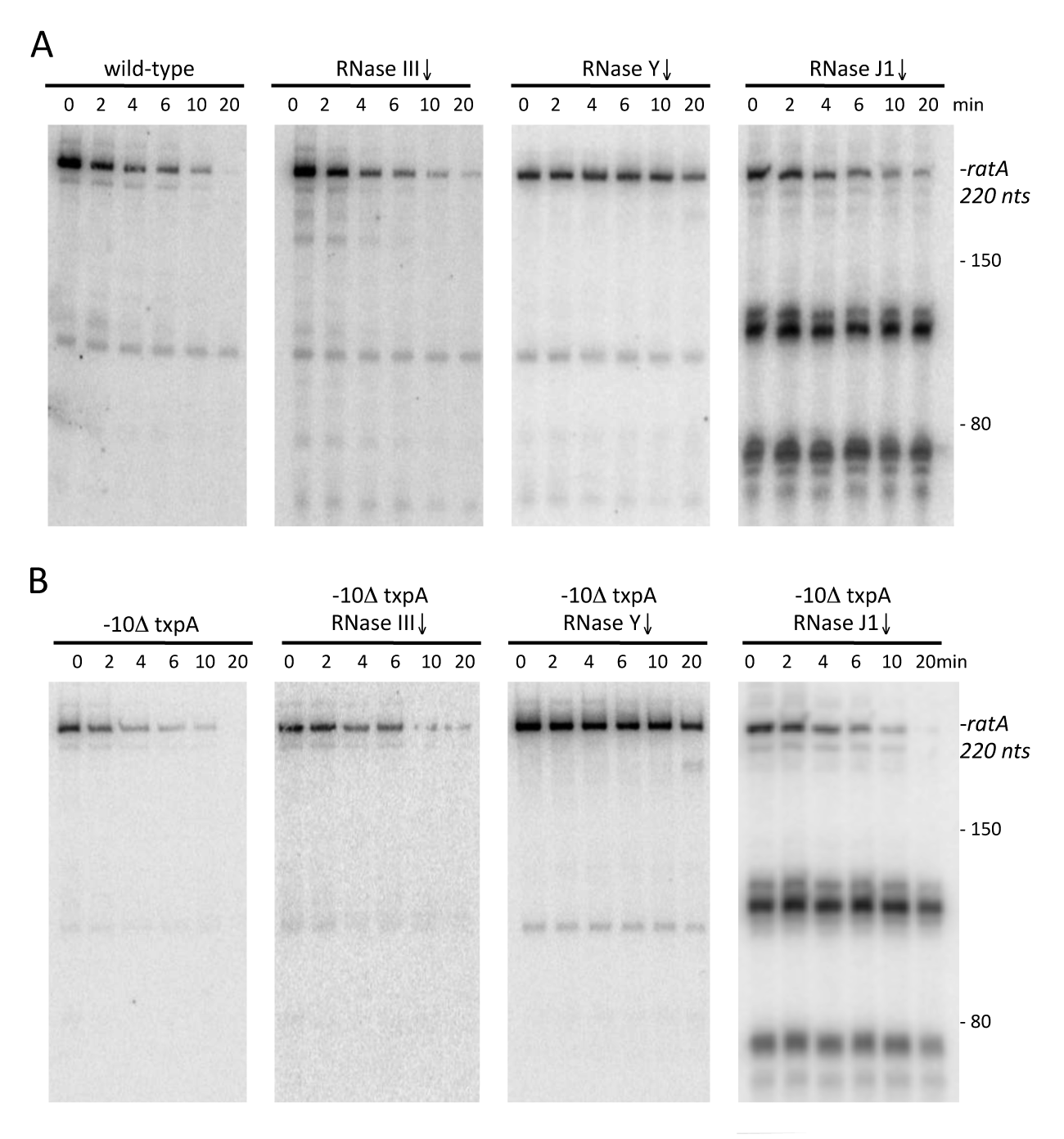

Supplement: Figure S4 — The degradation profile of RatA is identical in wild-type strains and in strains no longer expressing txpA. (A) High resolution (5% polyacrylamide) Northern blots performed on RNAs isolated from wild-type cells at times (min) after rifampicin addition (150 µg/ml) in strains depleted for RNase III (CCB288), RNase Y (CCB294) and RNase J1 (CCB034). Migration positions (in nts) of an RNA marker are given to the right of the figure. (B) High resolution Northern blots performed on RNAs isolated from txpA -10Δ cells (CCB325) and txpA -10Δ cells depleted for RNase III (CCB348), RNase Y (CCB338) and RNase J1 (CCB337). (TIF) [file pgen.1003181.s004.tif]

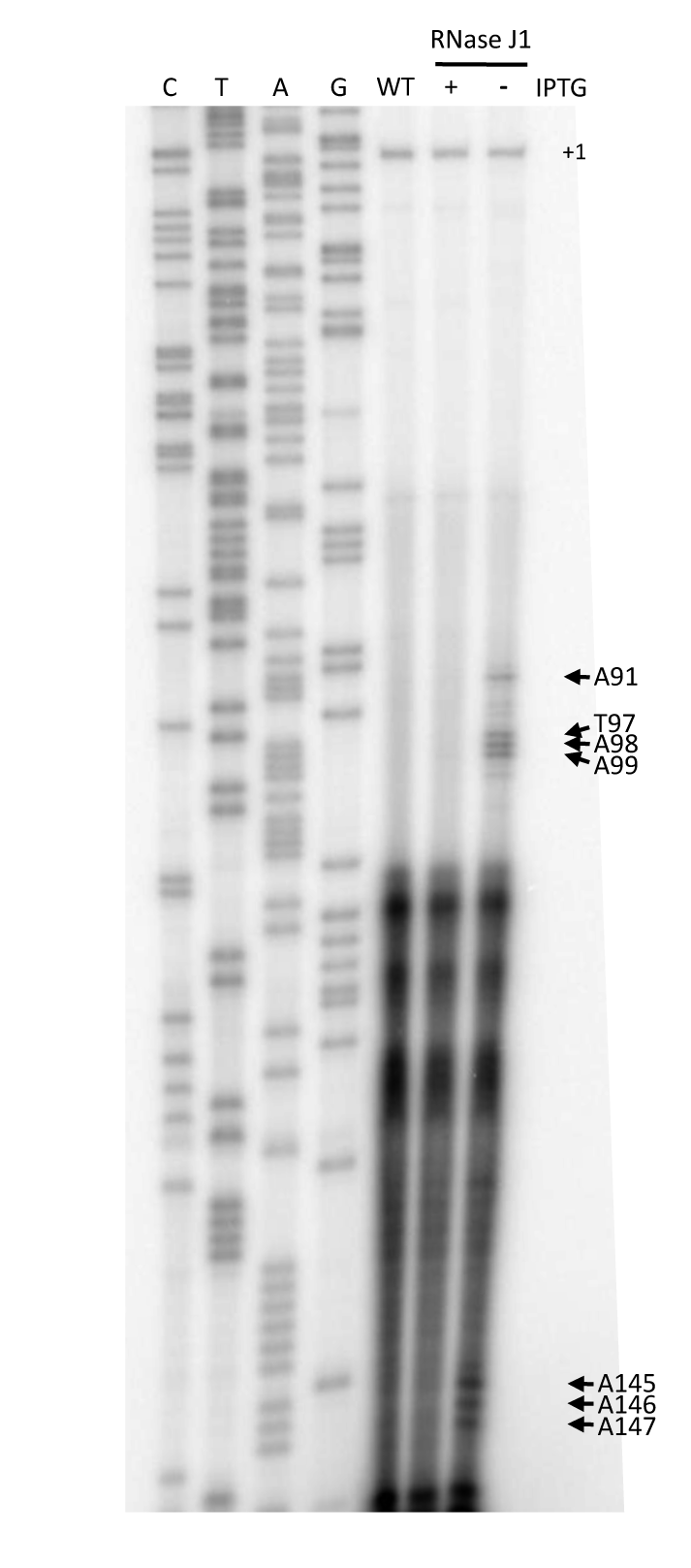

Supplement: Figure S5 — Mapping of 5′ ends of RatA intermediates that accumulate in strains depleted for RNase J1. Primer extension assay using oligo CC758 (Table S1) on 15 µg of total RNA isolated from wild-type strains (WT) and strain CCB034 (Table S2) grown in the presence and absence of IPTG. A sequence reaction performed with the same oligo on a PCR template of the txpA/RatA region (oligos CC795/796; Table S1) is shown to the left. The sequence is labeled as its reverse complement to facilitate direct reading. (TIF) [file pgen.1003181.s005.tif]

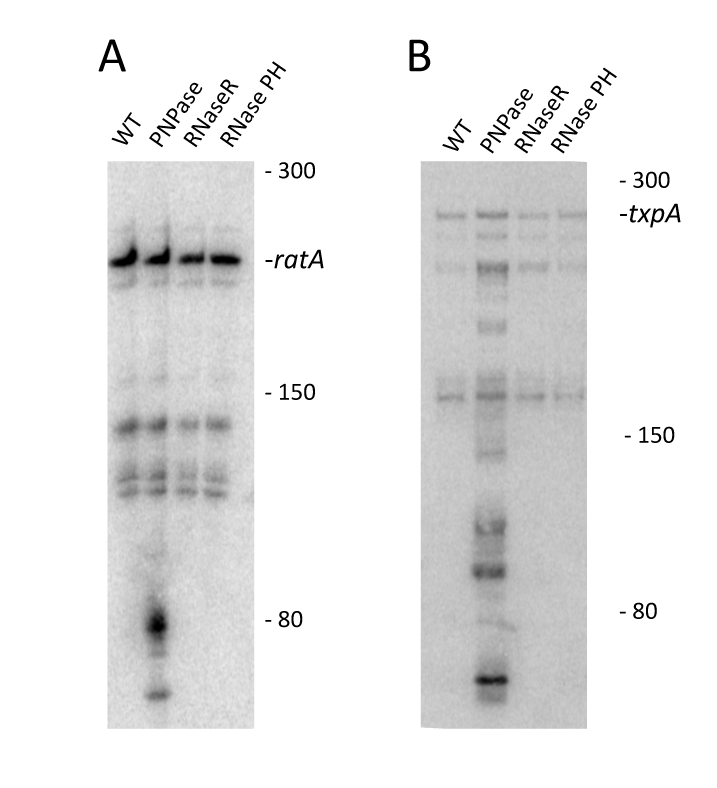

Supplement: Figure S6 — RatA and txpA degradation intermediates accumulate in the absence of PNPase. Northern blots of RNA isolated from wild-type (WT), PNPase (SSB1030), RNase R (CCB021) and RNase PH (CCB308) mutants (Table S2) probed with (A) oligo CC862 (Table S1) specific for the 5′ end of RatA and (B) oligo CC861 (Table S1) specific for the 5′ end of txpA. (TIF) [file pgen.1003181.s006.tif]

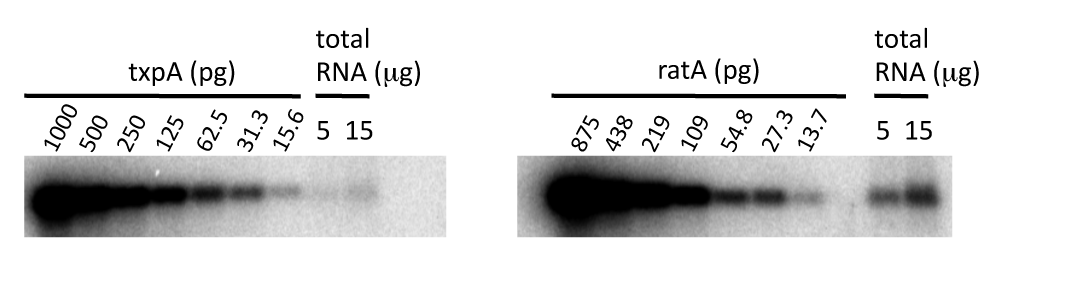

Supplement: Figure S7 — RatA is present in excess over txpA in wild-type cells. Quantitative Northern blot loaded with known quantities (in pg) of in vitro transcribed txpA and RatA RNAs, and either 5 or 15 µg of total RNA isolated from wild-type cells. (TIF) [file pgen.1003181.s007.tif]

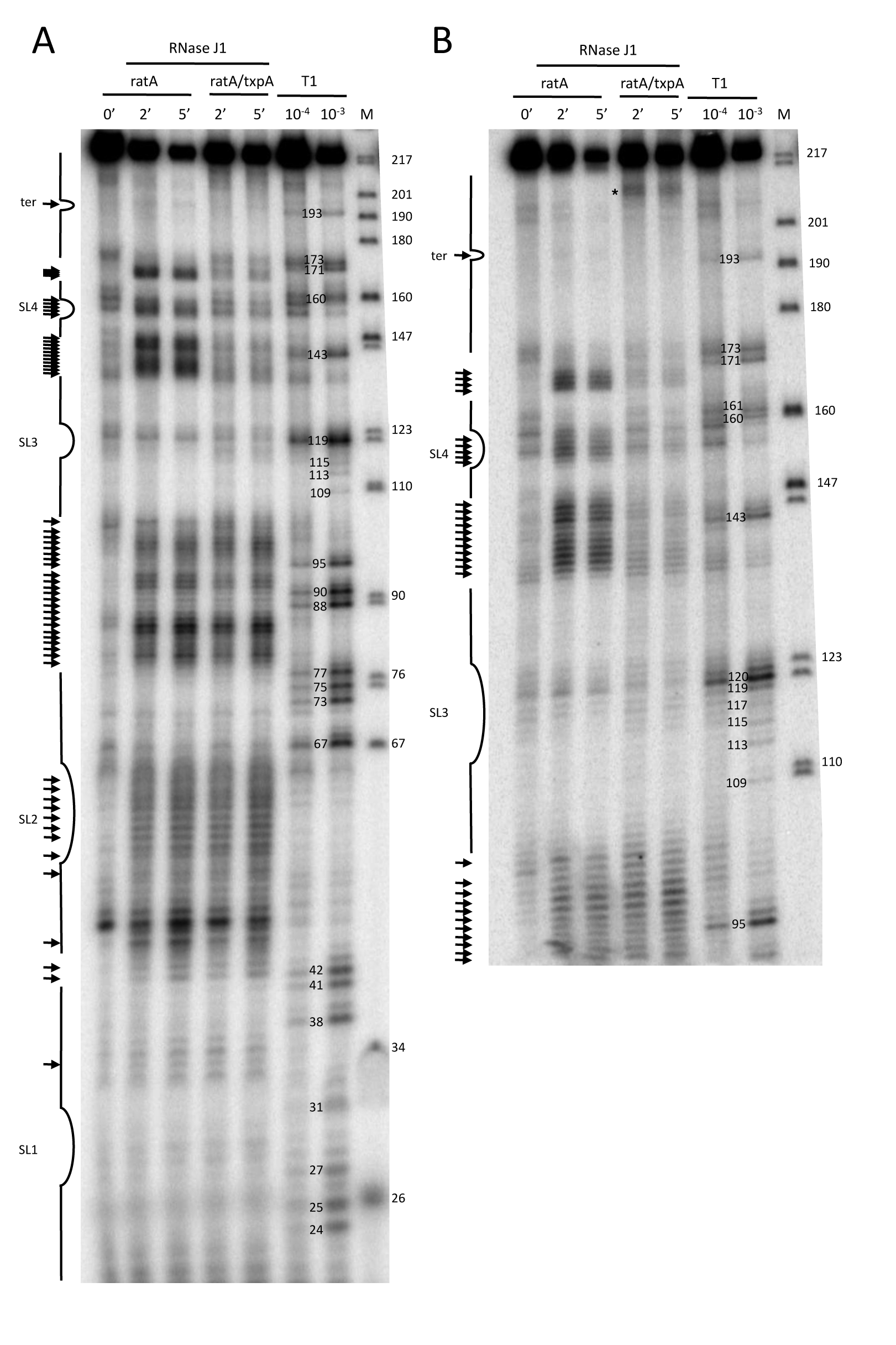

Supplement: Figure S8 — Structure probing of RatA RNA and RatA/txpA hybrid. In vitro transcribed 5′-labeled RatA RNA (0.5 pmol) alone hybridized to a 2-fold excess of unlabeled txpA were incubated with 0.6 µg RNase J1 for 2 or 5 minutes and loaded on a 5% polyacrylamide/urea gel. The RatA RNA was also digested with RNase T1 (Ambion) under denaturing conditions at the dilutions shown to reveal migration positions of G residues. A DNA size standard (in nts) is shown in the lane labeled M. (A) short migration (B) long migration with same samples. (TIF) [file pgen.1003181.s008.tif]

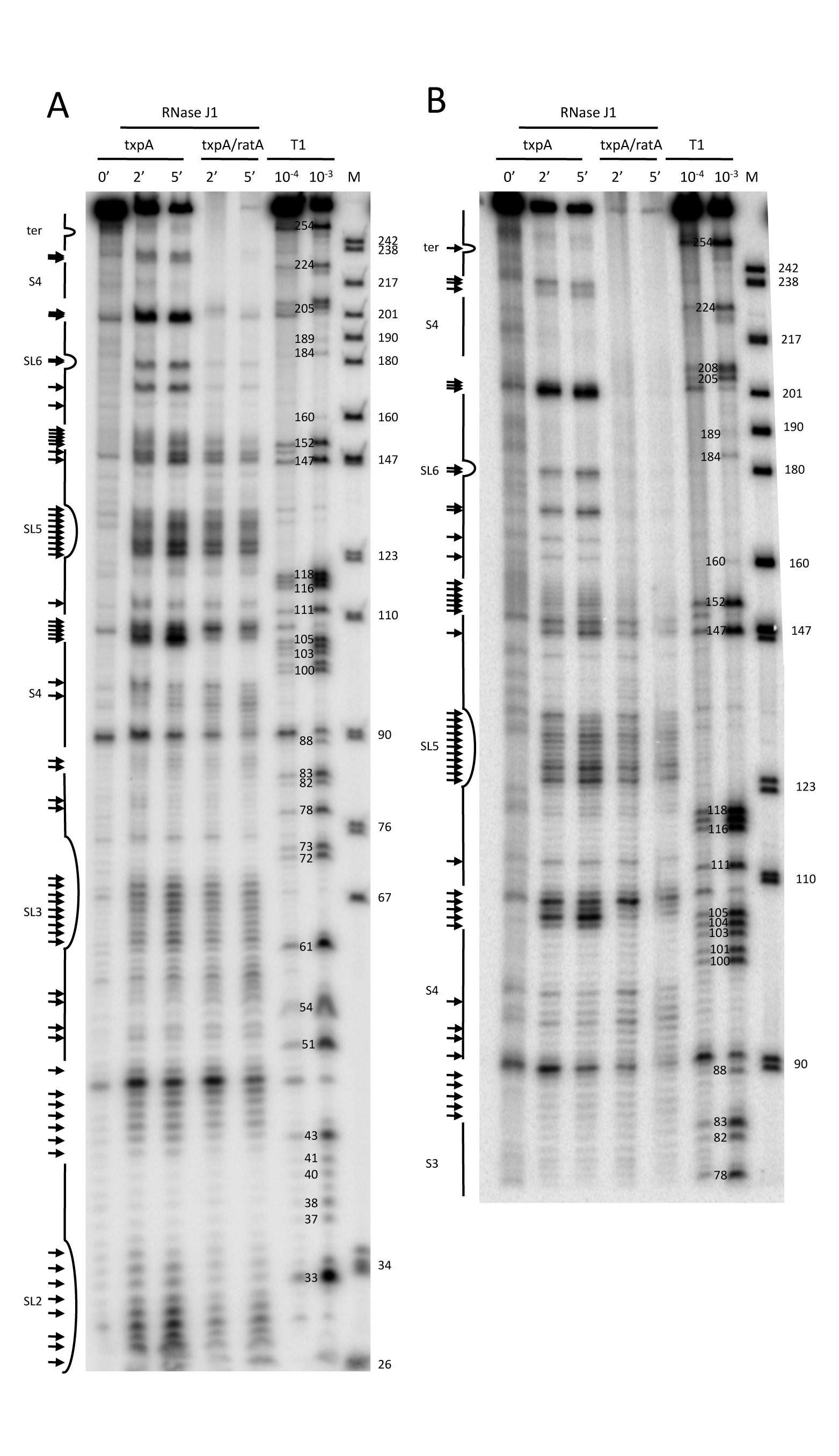

Supplement: Figure S9 — Structure probing of txpA RNA and txpA/RatA hybrid. In vitro transcribed and 5′ -labeled txpA RNA (0.5 pmol) alone or hybridized to a 2-fold excess of unlabeled RatA were incubated with 0.6 µg RNase J1 for 2 or 5 minutes and loaded on a 5% polyacrylamide/urea gel. The 5′ -labeled txpA RNA was also digested with RNase T1 (Ambion) under denaturing conditions at the dilutions shown to reveal migration positions of G residues. A DNA size standard (in nts) is shown to the right. (A) short migration (B) long migration with same samples. (TIF) [file pgen.1003181.s009.tif]

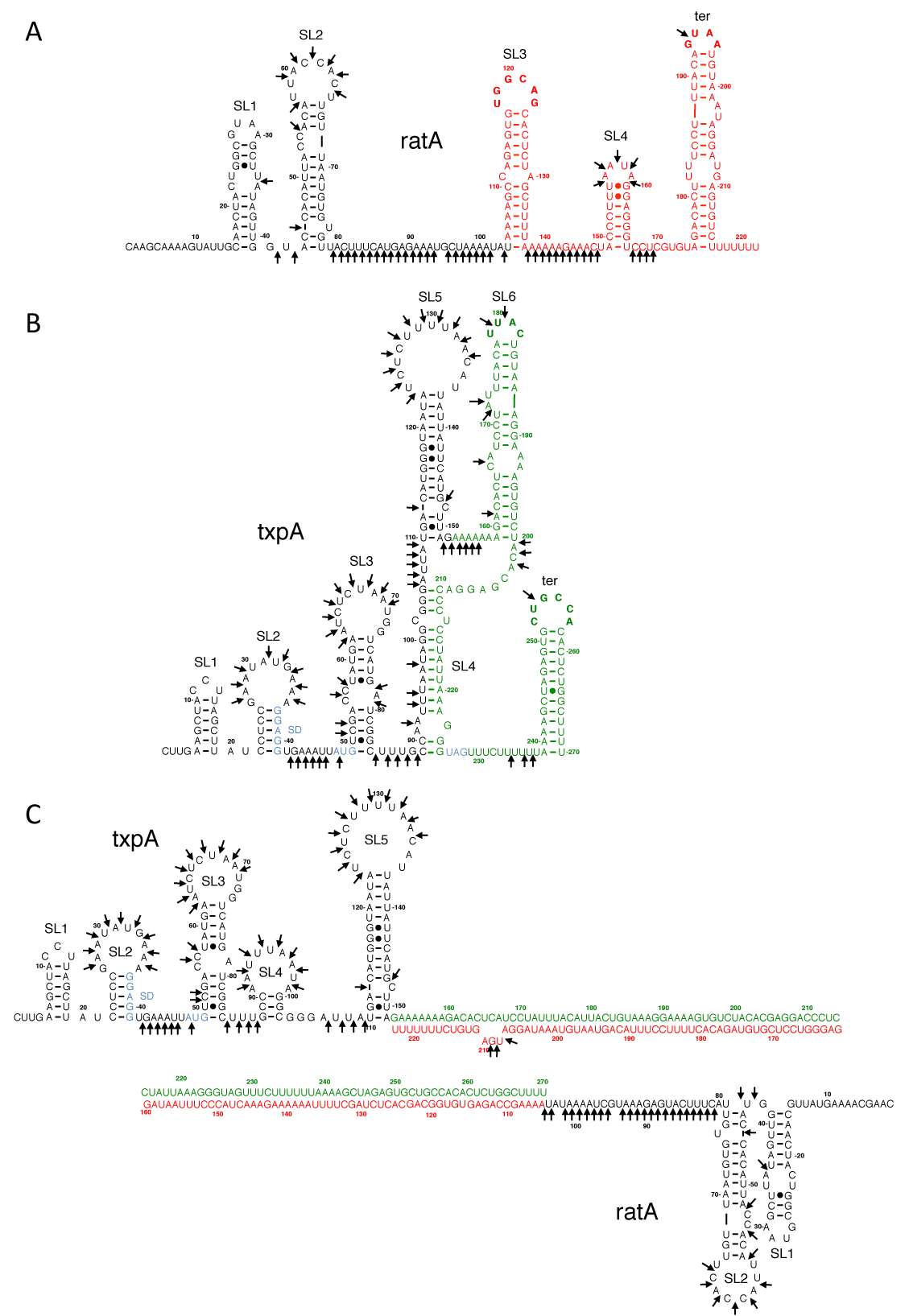

Supplement: Figure S10 — Summary of structure probing data for RatA, txpA and txpA/RatA hybrids. Mapped RNase J1 cleavages (arrowheads) on the best-fitting secondary structures of (A) RatA (B) txpA and (c) the txpA/RatA hybrid. Overlapping sequences of RatA and txpA are shown in red and green, respectively. The Shine-Dalgarno (SD) sequence, initiation and termination codons of txpA are shown in blue. (TIF) [file pgen.1003181.s010.tif]

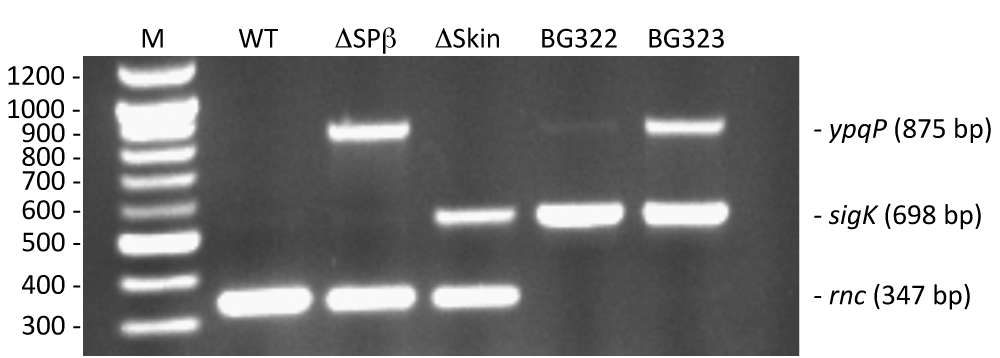

Supplement: Figure S11 — Suppressor strains BG322 and BG323 have excised the Skin prophage and have excised SPβ to different degrees. Agarose gel showing multiplex PCR analysis of rnc::spc suppressor strains. A PCR product corresponding to the reconstituted ypqP and sigK genes is indicative of excision of the SPβ and Skin prophages, respectively. Strains with a wild-type rnc gene give a 347 nt PCR fragment, while successfully deleted rnc strains do not give a PCR product. A DNA marker (bp) is shown in the lane labeled M. (TIF) [file pgen.1003181.s011.tif]
