# Supplementary material for: The Essential Function of B. subtilis RNase III Is to Silence Foreign Toxin Genes
Source: PLoS Genet. 2012 Dec 27;8(12):e1003181. doi: 10.1371/journal.pgen.1003181 (PMC3531473; doi:10.1371/journal.pgen.1003181)
Supplement: Table S1 — Oligonucleotides used in this study. Non-hybridizing sequences are in lower case letters. (DOC) [file pgen.1003181.s012.doc]

**Table S1** Oligonucleotides used in this study

| Oligo | Gene | Sequence |
| --- | --- | --- |
| CC435 | *rnc 5'* | gtatcatatgCATACGGCTTTTTCCGATGCTCATTC |
| CC657 | *ymdA* | atatatcatatgCGTAAAACCATTGCCGAAGCGAAAATTGCG |
| CC658 | *ymdA* | atatatggatccTTATTTTGCATACTCTACGGCTCGAGTCTCTCTG |
| CC756 | *as bsrH* | GAAGAAATAGACCCACCCCTTGAGCTCGGCAAAG |
| CC757 | *txpA 3'* | CTTTAATAGGAGGGTCCTCGTGTAGACACTTTTC |
| CC758 | *RatA 3'* | GAAAAGTGTCTACACGAGGACCCTCCTATTAAAG |
| CC759 | *bsrH* | CTTTGCCGAGCTCAAGGGGTGGGTCTATTTCTTC |
| CC795 | *yqbM* | cttgttactagtGCAAAACACCATTTCAGGTAAAGAGGGC |
| CC796 | *yqbN* | cttgttactagtGCTTTGTTCAGCCAATTTGCATATTCACC |
| CC816 | *rnc* | AAATTTACTAGTTAAGAGAAGCGCGGTGAACCTGATG |
| CC817 | *rnc* | atatatggatccGACGGCATACATACTGAAATC |
| CC861 | *txpA 5'* | GGCAAAGCCGATCATGACCATTAGAGATTC |
| CC862 | *RatA 5'* | GGTACCAACTATAAGCTTACGCCAGTAGTTGC |
| CC907 | *txpA* | gatgatggatccGCTTCTTCAAGGTCTTCGAGATCATCATC |
| CC908 | *txpA* | GATAAGCTAAGGTAGCTTCAAGAATCTCATGAATTATATGGAAAGCACATTC |
| CC909 | *txpA* | GAATGTGCTTTCCATATAATTCATGAGATTCTTGAAGCTACCTTAGCTTATC |
| CC910 | *txpA* | gatgatggatccGGCTTCTATGATCAGGTCATTTTGAACTCATAG |
| CC986 | *sigK 3'* | CGATAAAACTCATGAAACATCTTCATC |
| CC987 | *sigK 5'* | CTTAGAGCTCATGGCTAAAGGGGATG |
| CC990 | *ypqP 5'* | CGGGAGTCAAATTGTCAAACGCC |
| CC991 | *ypqP 3'* | GGCTTGATCTGATTGATACCCAGG |
| CC998 | *txpA* | GCTCTAATACGACTCACTATAGGGAAGCTACCTTAGCTTATCCTCCG |
| CC999 | *txpA* | AAAAGCCAGAGTGTGGCAGC |
| CC1000 | *ratA* | GCTCTAATACGACTCACTATAGGGCAAAAGTATTGCAACTACTGGCG |
| CC1001 | *ratA* | AAAAGACACTCATCCTATTTACATTAC |
| CC1008 | *as-yonT* | GCGGCTCCGACCAAAGAGACAACGCC |
| CC1011 | *rnc 3'* | GACGCTGTGAACTACATACAAAACCAGC |
| CC1015 | *bsrG/kan* | gtcttgctcatcccctatgaaaggggtgggaaaGAGGTGATAGGTAAGATTATACCGAGGTATGAAAACG |
| CC1016 | *kan/bsrG* | caagcaatagtattgcaaataccatttttatatggGCTTGTAGTTAAAGCTTTTTAGACATCTAAATC |
| CC1017 | *bsrG/kan* | CGTTTTCATACCTCGGTATAATCTTACCTATCACCTCtttcccacccctttcataggggatgagcaagac |
| CC1018 | *kan/bsrG* | GATTTAGATGTCTAAAAAGCTTTAACTACAAGCccatataaaaatggtatttgcaatactattgcttg |
| CC1019 | *yolA* | CGAGCTTTCACATATAGTTTATTCCCC |
| CC1020 | *yokL* | CCTCGTTCAGCTAATAACATGAGAGAATGGG |
| CC1071 | *yonS* | GTGACAGGCTTCTCGACGAGTACGTAGG |
| CC1072 | *yonT/ery* | GTTATAATAGAGTCATAGGAAAGGAGGTGTACATActttaactctggcaaccctcaaaattg |
| CC1073 | *yonT/ery* | caattttgagggttgccagagttaaagTATGTACACCTCCTTTCCTATGACTCTATTATAAC |
| CC1074 | *ery/yonT* | GGAGAAGTTCATTCCCCTTTAGCTTAGCTCgatagtttatggcggtgtagatgttgatg |
| CC1075 | *ery/yonT* | catcaacatctacaccgccataaactatcGAGCTAAGCTAAAGGGGAATGAACTTCTCC |
| CC1076 | *yonU* | GGAACGATAATTGCCTTACCGCTCCC |
| CC1101 | *yonT* | GTGCTTGAGAAAATGGGTATCG |
| CC1102 | *yonT(T7)* | gctctaatacgactcactatagggCATCGGCGTATACGTTGGCGTTGTCTC |
| CC1129 | *txpA* | CATTAGAGATTCATAGGTCGActtAATTTCACCTCCTTTCATATTCGG |
| CC1130 | *txpA* | CCGAATATGAAAGGAGGTGAAATTaagTCGACCTATGAATCTCTAATG |
| CC1147 | *ratA* | CGCCAGTAGTTGCAATACTTTTGCTTGGCACctttaactctggcaaccctcaaaattg |
| CC1148 | *ratA/ery* | caattttgagggttgccagagttaaagGTGCCAAGCAAAAGTATTGCAACTACTGGCG |
| CC1149 | *ery/ratA* | GGCTTCTATGATCAGGTCATTTTGAACTCgatagtttatggcggtgtagatgttgatg |
| CC1150 | *ratA/ery* | catcaacatctacaccgccataaactatcGAGTTCAAAATGACCTGATCATAGAAGCC |
| CC1151 | *ery/ratA* | CGACCTATGAATCTCTAATGGTCATG |
| CC1152 | *ratA* | GTGGTAAGATTACTTTAGAAGTAATTC |
| HP246 | 5S rRNA | ATCGGCGCTGAAGAGCTTAACTTCC |

Non-hybridizing sequences are in lower case letters
