# Supplementary material for: The Essential Function of B. subtilis RNase III Is to Silence Foreign Toxin Genes
Source: PLoS Genet. 2012 Dec 27;8(12):e1003181. doi: 10.1371/journal.pgen.1003181 (PMC3531473; doi:10.1371/journal.pgen.1003181)
Supplement: Table S2 — B. subtilis strains used in this study. (DOC) [file pgen.1003181.s013.doc]

Table S2. *B. subtilis* strains used in this study

| Strain | Genotype | Reference |
| --- | --- | --- |
| SSB1002 | W168 (trp prototroph) | Lab strain |
|  | 168 *trpC2* | [[30](#_ENREF_30)] |
|  | 168 *trpC2* ∆SPβ | [[30](#_ENREF_30)] |
| 7TFC7a | 168 *trpC2* ∆SPβ ∆Skin | [[30](#_ENREF_30)] |
| TF8a | 168 *trpC2* ∆SPβ ∆Skin ∆PBSX | [[30](#_ENREF_30)] |
| CCB021 | W168 *rnr::spc* | [[41](#_ENREF_41)] |
| CCB034 | W168 *rnjA*:pMUTIN-*rnjA* Ery | [[20](#_ENREF_20)] |
| CCB288 | W168 *rnc::spc* *amyE::Pspac-rnc* Cm | [[23](#_ENREF_23)] |
| CCB294 | W168 *rny::spc* *amyE::Pspac-rny* Cm | [[23](#_ENREF_23)] |
| CCB297 | W168 ∆Skin | This study |
| CCB302 | W168 *rnc::spc* *amyE*::pX-*rnc* Cm | This study |
| CCB308 | W168 *rph::spc* | This study |
| CCB325 | W168 *txp -10∆* | This study |
| CCB337 | W168 *txp -10∆ rnjA*:pMUTIN-*rnjA* Ery | This study |
| CCB338 | W168 *txp -10∆ rny::spc* *amyE::Pspac-rny* Cm | This study |
| CCB340 | W168 *txp -10∆ rnc::spc* *amyE::Pspac-rnc* Cm | This study |
| CCB361 | W168 *bsrG::kan* | This study |
| CCB363 | W168 SPβ::PIID-*sspB* kan | This study |
| CCB364 | W168 ∆Skin SPβ::PIID-*sspB* kan | This study |
| CCB365 | W168 *txp -10∆* SPβ::PIID-*sspB* kan | This study |
| CCB368 | W168 *txpA -10Δ bsrG::kan* | This study |
| CCB377 | W168 *sunA::kan* | This study |
| CCB402 | W168 *txpA -10Δ sunA::kan* | This study |
| CCB413 | W168 *yonT::ery* | This study |
| CCB414 | W168 *txpA -10Δ yonT::ery* | This study |
| CCB418 | W168 *txpA -10Δ yonT::ery rnc::spc* | This study |
| CCB422 | W168 ∆Skin SPβ::PIID-*sspB* kan *rnc::spc* | This study |
| CCB456 | W168 *txpA (AUG->AAG)* | This study |
| CCB461 | W168 *txpA (AUG->AAG)* P*ratA::ery* | This study |
| CCB467 | W168 *txpA (AUG->AAG) amyE::Pspac-rnc* Cm *rnc::spc* pMAP65 | This study |
| CCB468 | W168 *txpA (AUG->AAG)* P*ratA::ery amyE::Pspac-rnc* Cm *rnc::spc* pMAP65 | This study |
| BG322 | *rnc::spc sup1* | [[13](#_ENREF_13)] |
| BG323 | *rnc::spc sup2* | [[13](#_ENREF_13)] |
| SSB1030 | W168 *pnp::cm* | [[41](#_ENREF_41)] |
